# Chia seeds and coenzyme Q_10_ alleviate iron overload induced hepatorenal toxicity in mice via iron chelation and oxidative stress modulation

**DOI:** 10.1038/s41598-023-47127-3

**Published:** 2023-11-13

**Authors:** Shimaa A. Sadek, Mohamed Marzouk, Hanan R. H. Mohamed, Bassant F. Abd El-sallam, Abdo A. Elfiky, Amany A. Sayed

**Affiliations:** 1 Zoology Department, Faculty of Science, Cairo University, Giza, Egypt; 2 Biophysics Department, Faculty of Science, Cairo University, Giza, Egypt

**Keywords:** Molecular biology, Health occupations, Risk factors

## Abstract

Iron overload (IOL) can cause hepatorenal damage due to iron-mediated oxidative and mitochondrial damage. Remarkably, combining a natural iron chelator with an antioxidant can exert greater efficacy than monotherapy. Thus, the present study aimed to evaluate the efficacy of Chia and CoQ_10_ to chelate excess iron and prevent hepatorenal oxidative damage in IOL mice. Male Swiss albino mice (*n* = 49) were randomly assigned to seven groups: control, dietary Chia, CoQ_10_, IOL, IOL + Chia, IOL + CoQ_10_, and IOL + Chia + CoQ_10_. Computational chemistry indicates that the phytic acid found in the Chia seeds is stable, reactive, and able to bind to up to three iron ions (both Fe^2+^ and Fe^3+^). IOL induced a significant (P < 0.05) increase in serum iron, ferritin, transferrin, TIBC, TSI, RBCs, Hb, MCV, MCH, WBCs, AST, ALT, creatinine, and MDA. IOL causes a significant (P < 0.05) decrease in UIBC, platelets, and antioxidant molecules (GSH, SOD, CAT, and GR). Also, IOL elicits mitochondrial membrane change depolarization, and DNA fragmentation and suppresses mitochondrial DNA copies. Furthermore, substantial changes in hepatic and renal tissue, including hepatocellular necrosis and apoptosis, glomerular degeneration, glomerular basement membrane thickening, and tubular degeneration, were observed in the IOL group. Dietary Chia and CoQ_10_ induced significant (P < 0.05) amelioration in all the mentioned parameters. They can mostly repair the abnormal architecture of hepatic and renal tissues induced by IOL, as signified by normal sinusoids, normal central veins, and neither glomerular damage nor degenerated tubules. In conclusion, the combined treatment with Chia + CoQ_10_ exerts more pronounced efficacy than monotherapy in hepatorenal protection via chelating excess iron and improved cellular antioxidant status and hepatorenal mitochondrial function in IOL mice.

## Introduction

Although iron is essential for vital functions in the body, and its insufficiency causes anemia and other health problems, its excess is unfavorable to normal cells because human beings lack the effective mechanism to remove excess iron^1,2^. Excess free iron can potentially promote cellular damage and alter vital organelle integrity by generating reactive oxygen species (ROS), mainly reactive hydroxyl radicals, via catalyzing Haber–Weiss reduction followed by Fenton reactions^3^. Furthermore, excess fee iron disrupts iron hemostasis and excessive free iron deposition in parenchymal organs, ultimately resulting in iron overload syndrome (IOL)^4^. IOL is greatly exacerbated by (1) Genetic defects in iron absorption, especially resulting from hereditary hemochromatosis in which increased dietary iron absorption into the duodenal enterocytes; (2) Frequent parenteral iron administration in transfusion-dependent anemia; (3) Pathological ailments characterized by mild iron deposition or dysregulation of body iron distribution^5,6^. Also, IOL is relatively common in some diseases, including alcoholic liver disease, hepatosteatosis, hepatitis C, hepatic cirrhosis, and hepatocellular carcinoma^7^. Iron overload causes hepatic damage, as the liver takes up the excessive iron from the blood and deposits it in the hepatic parenchyma and reticuloendothelial cells^8^. Iron not only exerts its toxic effect on hepatic tissue but also enhances renal tubular damage and may disturb physiological renal function. As the kidney has an adequate amount of mitochondria, a major source of oxidative stress, it is highly vulnerable to oxidative stress induced by excess iron^9^.

Commercially, deferoxamine, deferasirox, and deferiprone, available synthetic ion-chelating compounds, are used to remove excess iron from the bloodstream via urine or feces by forming complexes^10^. Although these compounds are effective in the treatment of IOL syndrome, their uses are associated with several limitations, particularly low oral bioavailability and/or limited plasma half-life, which make them suboptimal^11^. Furthermore, their efficacy is limited because they cannot halt iron-mediated oxygen reduction. Therefore, bioactive compounds that can trap free radicals may be considered iron chelators and used as iron-removable therapy instead of conventional iron chelators.

Chia, *Salvia hispanica*, is an essential part of the human diet because of its extraordinary nutritional value and health-promoting properties. This study selects Chia seeds as they contain a considerable amount of phytic acid, a potent metal phytochelator, besides its contents of phenolic compounds, omega-3-linolenic acid, fibers, and minerals^12^. Thus, Chia seeds may be potentially utilized as a promising natural iron phytochelator for IOL treatment. Additionally, utilizing natural antioxidants is a potential strategy for IOL management via attenuation of ROS generation^13^. Coenzyme Q_10_ (CoQ_10_), also known as ubiquinone, is an endogenous lipophilic antioxidant compound that presents mainly in the mitochondrial membrane of all types of human cells^14^. During the respiratory chain, CoQ_10_ is reduced to its active ubiquinol form, an effective antioxidant, which prevents both initiation and propagation of lipid peroxidation^15^. The CoQ_10_ supplement could be effectively used as a potential remedy for IOL due to its ability to quench singlet oxygen and peroxides.

Computational chemistry has been successful in the past few decades in studying the biological significance of compounds, including natural-based compounds, and understanding the mechanism of action of many molecules^16,17^. Therefore, it could be informative to know the stability and reactivity of molecules and predict their biological function before the experimental validation. Thereby, the present study aimed to evaluate the ameliorating efficacy of Chia seeds and CoQ_10_ on hepatorenal disorders associated with iron overload-induced toxicity in mice. Additionally, the physicochemical properties of the main component in Chia seeds, phytic acid, CoQ_10_, and deferoxamine, were tested using computational chemistry tools.

## Materials and methods

### Computational calculations

The PubChem database (https://pubchem.ncbi.nlm.nih.gov/) was utilized to obtain the starting structures (2D) of phytic acid (CID: 890), CoQ_10_ (CID: 5281915), and deferoxamine (CID: 2973) in structure data file (SDF) format^18^. After that, the computational chemistry platform SCIGRESS 3.0 was utilized to minimize and calculate the molecular properties of the three compounds^19^. The geometry optimization was performed in two steps: classically, using the MM3 force field, and then by the semi-empirical parameterization method 6 (PM6) in water^20,21^. The optimized structures were ensured by calculating their infrared (IR) absorption peaks using the PM6 force. Now, the structures are ready for parameter calculations.

Different parameters and properties were calculated from the optimized compounds, including conformation minimum energy (kcal/mol), dipole moment (debye), pKa, electron affinity (eV), dielectric energy (kcal/mol), steric energy (kcal/mol), total energy (kcal/mol), the heat of formation (kcal/mol), ionization potential (eV), Highest occupied molecular orbital (HOMO) (eV), Lowest unoccupied molecular orbital (LUMO) (eV), logP, molar refractivity, polarizability (Å^3^), molecular weight, solvent accessible surface area (Å^2^), Hydrogen bond donor and acceptor counts, the role of five violations^16^. Additionally, some thermodynamics information was calculated for each compound, which includes free energy (kcal/mol), enthalpy (kcal/mol), entropy (cal/mol Kelvin), heat capacity (cal/mol Kelvin), and heat of formation (kcal/mol).

### Chemicals and preparation

Coenzyme Q_10_ (CoQ_10_) was procured from Sigma-Aldrich (St. Louis, MO, USA). As specified by the supplier, CoQ_10_ is a yellow crystalline powder with a purity of 99% based on HPLC analysis. Before administration, its suspension was prepared in saline (0.9% NaCl). The Chia seeds (*Salvia hispanica* L.) were purchased from a local market (Giza, Egypt). The seeds were cleansed by eliminating the impurities. After that, the seeds were wrinkled, then their flour was filled in polyethylene aluminum bags and kept at – 20 °C till use. Haemojet ampoules were purchased from a local pharmacy; the European Egyptian Pharmaceutical Industrial Company, Alexandria, Egypt, produces the drug. Each ampoule has 100 mg of elemental iron in the form of a ferric hydroxide poly maltose complex. All other chemicals and reagents used were of the highest purity.

### Evaluation of in vitro iron-chelating efficacy of Chia and CoQ_10_

Iron chelation potency of both Chia and CoQ_10_ was achieved according to the method of Khalili et al.^22^. Concisely, 0.5 ml of 2 mM FeCl_2_ was added to 1 ml of each Chia, CoQ_10_, and EDTA (standard metal chelator) at various concentrations (50–400 µg/ml). The reaction was started by the addition of 5 mM ferrozine (0.2 ml). Then, the mixture was shaken strongly and kept at room temperature for 15 min in the dark. After incubation, the absorbance of each solution was measured at a wavelength (λ) of 562 nm against distilled water as blank. The control tube (containing all reagents only) was prepared similarly. The percentage of iron chelation potency was calculated by applying the following equation:

$$ \% {\text{ Iron chelation potency}}\, = \frac{{\left[ {{\text{A}}_{{{\text{Control}}}} - {\text{A}}_{{{\text{Sample}}\;{\text{or}}\;{\text{Standard}}}} } \right]}}{{{\text{A}}_{{{\text{Control}}}} }} \times 100 $$

where A _control_ is the absorbance of the control and A _sample_ is the absorbance of Chia/ CoQ_10_. Then, the half-maximal effective concentration (EC_50_) was calculated according to an equation from the plot graph between the percentage of iron-chelating activity and the different sample concentrations.

### Experimental animals

Adult male Swiss albino mice (*Mus musculus*), weighed 20‒25 g were utilized in this study. Animals were obtained from the National Research Center (NRC) in Egypt. They were divided (seven animals/in each cage) and maintained in polypropylene cages bedded with sawdust. They were housed in a standard animal house condition (23 ± 2 °C) in a natural day/night cycle with chow pellets feeding and drinking water ad libitum*.* Animals were adapted to the new animal house conditions for seven days before the commencement of the experiment. All experimental procedures performed were approved by the Cairo University, Institutional Animal Care and Use Committee (IACUC) (Egypt) (Approval No. CU/I/F/87/17). All the experimental procedures were carried out in agreement with the requirements of the Guide for the Care and Use of Laboratory Animals 8th Edition 2011 (the Guide).

### Preparation of diet containing Chia seeds

The diet containing Chia seeds, *Salvia hispanica*, was prepared according to Chicco et al.^23^. The whole diet contained a standard powdered commercial diet and Chia seeds flour (362 g/kg diet), where the composition of the rodent commercial diet is 48% carbohydrate, 27% proteins, 7% fat, 8% fiber, 9% minerals, and vitamins as stated by the manufacture. To prepare the diet with Chia seeds flour, 100 g of powdered commercial chow was well mixed with 36.2 g of Chia seeds flour until forming a homogenous dough-like texture.

### Iron overload (IOL) syndrome induction

Iron overload syndrome is induced by intraperitoneal injection of ferric hydroxide poly maltose complex (IPC) using haemojet ampoules (3 doses/week) at a dosage of 50 mg/kg body weight for 4 subsequent weeks^24^.

### Experimental design and treatment

Forty-nine adult Swiss male mice, aged 6–8 weeks and weighing 23 ± 2 g were randomly allocated into 7 groups (seven mice/group) and treated as follows:

- Group 1: Control; mice were administered vehicle (saline) daily for 4 weeks.
- Group 2: Chia-containing diet; mice fed daily with ground Chia-containing diet for 4 weeks^25^.
- Group 3: CoQ_10_; mice were administered CoQ_10_ orally at 300 mg/kg b.wt for 4 weeks.
- Group 4: IOL; mice intraperitoneally injected ferric hydroxide polymaltose complex (IPC) (3 doses/ week) at 50 mg/kg b.wt for 4 weeks.
- Group 5: IOL + Chia; mice treated concurrently with IPC (3 doses/ week) at 50 mg/kg b.wt and Chia diet for 4 weeks.
- Group 6: IOL + CoQ_10_; mice treated concurrently with IPC (3 doses/ week) at 50 mg/kg body weight and CoQ_10_ (300 mg/kg b.wt) daily for 4 weeks.
- Group 7: IOL + Chia + CoQ_10_; mice treated concurrently with IPC (3 doses/ week) at 50 mg/kg b.wt and Chia diet and CoQ_10_ (300 mg/kg b.wt) for 4 weeks.

During the experimental period, groups 1, 3, 4, and 6 were fed a daily commercial diet consisting of 48% carbohydrates, 27% proteins, 7% fat, 8% fiber, and 9% minerals and vitamins.

### Animal handling

The food intake was checked daily during the experimental period, whereas the body weight was recorded weekly. At the termination of the experimental period, the mice were fasted overnight and euthanized with an overdose of sodium pentobarbital. Blood was collected in a centrifuge and heparinized tubes to assess some hematological and hepatorenal biomarkers. Sera were obtained by centrifugation (3000 rpm, 10 min.) and stored at − 20 °C until used. Liver and kidney samples were immediately harvested and weighed to calculate their relative weight. Then, the samples were washed with saline and used to assess the oxidative stress markers, some molecular assays, and histological study.

### Assessment of serum iron indices

Iron concentration and total iron-binding capacity (TIBC) were estimated in serum using spectrum kits according to the methods adopted by Stookey^26^ and Viollier et al.^27^. Serum ferritin and transferrin levels were determined using the sandwich enzyme-linked immunoassay method (ELISA) according to the manufacturer’s instructions. Furthermore, serum transferrin saturation index (TSI) was calculated according to the following equation: TSI% = [(Serum iron/TIBC) × 100]. Also, the unsaturated iron-binding capacity (UIBC) was calculated based on the following equation: UIBC = [TIBC − Serum iron]^28^.

### Hematological assessment

A hematological auto-analyzer (BC-2800 VET Mindray auto hematology analyzer, USA) was used to determine total red blood cells (RBCs), hemoglobin (Hb) content, hematocrit (HCT), mean corpuscular volume (MCV), and mean corpuscular hemoglobin (MCH). Furthermore, the total WBCs and platelets count was determined.

### Assessment of hepatic and renal function markers

Serum aminotransferase enzyme (AST & ALT) activities were assessed using Spectrum kits based on the colorimetric methods designated by Breuer^29^. Also, total protein and albumin concentrations were measured using Biodiagnostic kits according to Henry et al.^30^ and Doumas et al.^31^, respectively. Serum creatinine as a specific renal function marker was determined spectrophotometrically using a commercial kit (Biodiagnostic, Giza, Egypt), according to Bartles et al.^32^.

### Determination of oxidative and antioxidative markers

Firstly, liver and kidney tissues were homogenized (10% w/v) in an ice-cold Tris HCl buffer (0.1 M, pH 7.4) and centrifuged at 3000 rpm/min for 15 min. Then, the obtained hepatic and renal supernatant was used to determine malondialdehyde (MDA), glutathione reduced (GSH), superoxide dismutase (SOD), catalase (CAT) and glutathione reductase (GR) using the commercial kits.

### Assessment of mitochondrial membrane potential change (MMP, Δψ)

The mitochondrial membrane potential in the hepatocyte and renal cells was determined based on the preferential staining of intact mitochondria with the fluorescent dye Rhodamine-123. Firstly, a small portion of the hepatic and renal tissues was grinded gently in phosphate buffer saline (PBS). Then, cell suspension was mixed with Rhodamine 123 (10 mg/ml) dye and incubated for 30 min at 37 °C away from light. Subsequently, stained cells were washed with PBS twice and finally the emitted fluorescent light from stained mitochondria was photographed at 200× magnification by an epi-fluorescent microscope (OLYMPUS CKX 41)^33^.

### Determination of DNA fragmentation

The apoptotic fragmentation genomic DNA in the hepatic and renal tissues was assessed using the protocol previously described by Gercel-Taylor^34^. About 60 mg of hepatic or renal tissues was minced and lysed in cold lysis buffer and then, the samples were centrifuged. Tris–EDTA buffer containing trichloroacetic acid (TCA) was then added to both cell pellets and supernatants, left for 20 min. at room temperature and centrifuged. The resultant precipitates were resuspended in 5% TCA, boiled for 15 min, and centrifuged again. Finally, diphenylamine was added to the clear supernatants, left overnight in darkness at room temperature, and measured the absorbance of the resultant color using a spectrophotometer at a wavelength of 600 nm. The amount of fragmentized DNA is expressed as a percentage of the total DNA.

### Quantification of mitochondrial DNA copies

To measure the number of hepatic and renal mitochondrial DNA copies the total RNA was first extracted from hepatic and renal tissues using the Gene JET RNA Purification kit (Thermo-scientific, USA), converted the extracted RNA into complementary DNA (cDNA) based on the manufacturer instructions of Revert Aid First Strand cDNA Synthesis kit (Thermo-scientific, USA). Finally, quantitative Real-Time Polymerase Chain Reaction (qRT-PCR) was done to quantify and amplify the mitochondrial gene-encoded 12S ribosomal RNA (12S rRNA) using SYBER Green master mix and the sequences of the primers displayed in Table 1^34^. The amplification of the mitochondrial encoded 12S rRNA gene was standardized against the amplification of the housekeeping nuclear 18S rRNA gene. The copies number of mitochondrial DNA was assessed using the relative comparative Ct (DDCt) method.

**Table 1:** Sequences of the primers used in RT-PCR.

Gene	Strand	Sequence
12S rRNA	Forward	5-ACCGCG GTC ATA CGA TTA AC-3
	Reverse	5-CCC AGTTTG GGT CTT AGC TG-3
18S rRNA	Forward	5-CGC GGTTCT ATT TTG TTG GT-3
	Reverse	5-AGT CGG CATCGT TTA TGG TC-3

### Histological examination

Hepatic and renal tissues were washed in saline and fixed in neutral buffered formalin (10%, pH 7.0) for 24 h. Then, tissues were processed as the standard methods to obtain paraffin sections, then cut, and stained with hematoxylin–eosin (H&E) for examination.

### Statistical analysis

The present results were displayed as means ± standard error of mean (SEM) of seven animals. One-way analysis of variance (ANOVA) followed by the Duncan test was utilized to compare between groups using SPSS (SPSS Inc., Chicago, IL, USA) software. Values of P < 0.05 were considered as statistically significant. The percentage of improvement was used to compare the ameliorative efficacy of monotherapy (Chia or CoQ_10_) and combined therapy (Chia + CoQ_10_).

### Ethical approval

All experimental procedures performed were reported according to ARRIVE guidelines and approved by the Cairo University, Institutional Animal Care and Use Committee (IACUC) (Egypt) (Approval No. CU/I/F/87/17). All the experimental procedures agreed with the requirements of the Guide for the Care and Use of Laboratory Animals 8th Edition 2011 (the Guide).

## Results

### Computational calculations

#### The structure of phytic acid, CoQ_10_, and deferoxamine

**Figure 1:**
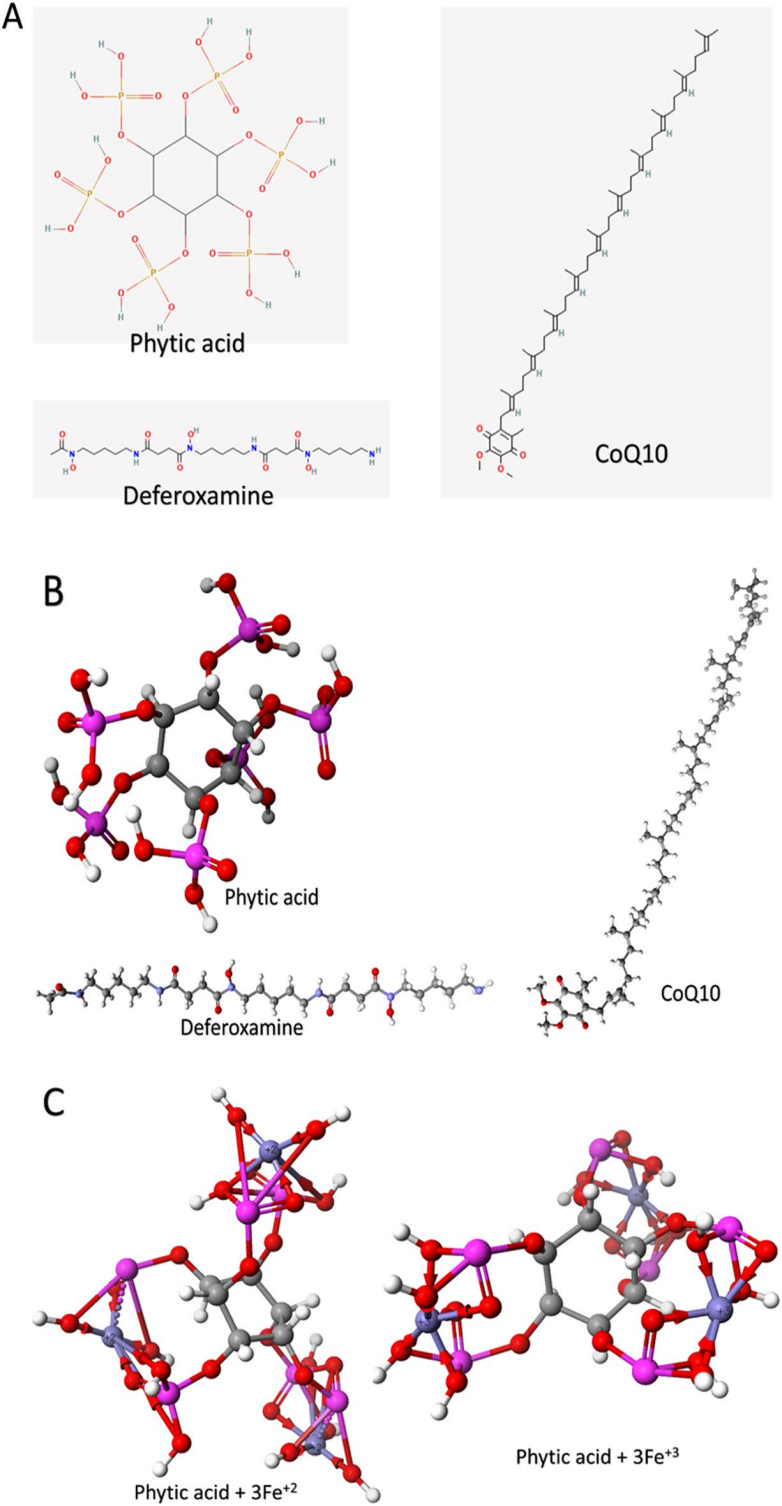
The structure of phytic acid, CoQ_10_, and deferoxamine in 2D (**A**) and 3D (**B**). The optimized formed complexes of phytic acid with Fe^2+^ and Fe^3+^ calculated at the PM6 level of theory (**C**). Atoms are colored according to this scheme; Carbon: gray, Oxygen: red, Nitrogen: violet, Phosphorus: pink, Iron: purple, and Hydrogen: white.

The 2D structures of phytic acid, CoQ_10_, and deferoxamine are shown in Fig. 1. The optimized structures are used to calculate the infrared (IR) spectrum using the same computational level (semi-empirical parameterization method 6). These spectra were calculated to ensure that the compounds are optimized at the PM6 level of theory^35^. All three compounds' IR spectra are positive, indicating good geometry optimization at the PM6 level of theory. The 2D and 3D of the three compounds are shown in Fig. 1A,B, respectively. Figure 1C shows the optimized complexes of phytic acid with both Fe^2+^ and Fe^3+^ at the PM6 level of semi-empirical QM level. Atoms are colored according to this scheme: Carbon: gray, Oxygen: red, Nitrogen: violet, Phosphorus: pink, Iron: purple, and Hydrogen: white. As shown in Fig. 1C, one phytic acid molecule can bind to up to three iron ions stably and could be a suitable natural iron chelator inside the cell.

#### Quantitative Structure–Activity Relationship (QSAR) parameters for phytic acid, CoQ_10_, and deferoxamine

The calculated parameters are used to compare phytic acid, CoQ_10_, and deferoxamine to understand their stability and the proposed activity. Table 2 lists the computed parameters for the three compounds. Some parameters reflect the molecule's stability, such as the minimum conformation (total) energy, the dielectric energy, and ionization potential. In contrast, other parameters reflect the reactivity of the molecule, such as the number of Hydrogen-bond acceptors and donors, dipole moment, electron affinity, the energy gap (difference between HOMO and LUMO), the partition coefficient (Log P), molar refractivity, and the solvent accessible surface area. All the compounds have molecular weight < 500 gm/mol, which slightly exceeds the limit of the role of five, but deferoxamine possesses the best value (560.684 g/mol) compared to the phytic acid (660.035 g/mol) and CoQ_10_ (863.343 g/mol).

**Table 2:** Quantitative Structure–Activity Relationship (QSAR) parameters calculated for phytic acid, CoQ_10_, and deferoxamine using semi-empirical Parameterization Method 6 (PM6).

Compound	Conformation minimum energy [kcal/mol]	Dielectric energy [kcal/mol]	Ionization potential [eV]	H-bond donors count	H-bond acceptors count	Dipole moment [debye]	Electron affinity [eV]	Energy gap (LUMO–HOMO) [eV]	Log P	Molar refractivity	Molecular weight [g/mol]	Solvent accessible surface area [Å^2^]
Phytic acid C_6_ H_18_ P_6_ O_24_	**− 1,462,194**	**− 3167**	**− 11.567**	**12**	**24**	0.398	0.922	10.645	**2.303**	**97.571**	660.035	406.562
CoQ_10_ C_59_ H_90_ O_4_	− 164,318	− 1199	− 9.065	0	4	**5.206**	**1.601**	**7.464**	15.272	284.210	863.343	**1079.732**
Deferoxamine C_25_ H_48_ N_6_ O_8_	− 320,689	− 2692	− 9.455	7	14	1.861	− 0.025	9.480	**− 1.000**	144.088	560.684	671.016

The best values are in bold, as illustrated in the text.

Phytic acid possesses the best stability parameters compared to the other two molecules. It has the lowest conformation minimum energy (− 1,462,194 kcal/mol) and the lowest dielectric energy (− 3167 kcal/mol) as demonstrated in Table 2. Regarding the reactivity of compounds, again, phytic acid shows the largest number of Hydrogen-bond donors (12) and acceptors (24), the lowest ionization potential (− 11.567 eV), and the lowest molar refractivity value (97.571) compared to deferoxamine and CoQ_10_. On the other hand, CoQ_10_ shows the highest dipole moment (5.206 debyes), highest electron affinity (1.601 eV), lowest LUMO–HOMO energy gap (7.464 eV), and the largest solvent-accessible surface area (1079.732 Å^2^) compared to phytic acid and deferoxamine. CoQ_10_ has no H-bond donors, only 4 H-bond acceptors, and an extremely high log P (15.272) value, reflecting the highest hydrophobicity. In addition, it possesses the highest molecular weight limiting its pharmacological properties compared to the other two compounds.

#### Thermodynamics properties of phytic acid, CoQ_10_, and deferoxamine

Table 3 demonstrates that phytic acid has the best thermodynamic properties as compared to CoQ_10_ and deferoxamine. Phytic acid has the lowest average free energy (− 1,228,071 ± 63,141.3 kcal/mol), lowest average enthalpy (36,340 ± 16,529.9 kcal/mol), lowest average entropy (267,158 ± 47,749.6 cal/mol/Kelvin), the lowest average heat capacity (169,495 ± 28,600.5 cal/mol/Kelvin), and the lowest average heat of formation (− 1,453,309 ± 16,529.7 kcal/mol). On the other hand, CoQ_10_ displays the highest free energy (197,463 ± 96,848.4 kcal/mol), highest average enthalpy (70,613 ± 32,219.7 kcal/mol eV), highest average entropy (556,608 ± 91,694.3 cal/mol/Kelvin), the highest average heat capacity (329,468 ± 75,736 cal/mol/Kelvin), and the highest average heat of formation (− 146,475 ± 32,221.3 kcal/mol) when compared with phytic acid and deferoxamine.

**Table 3:** Thermodynamics properties of phytic acid, CoQ_10_, and deferoxamine calculated using semi-empirical Parameterization Method 6 (PM6).

Compound	Free energy [kcal/mol]	Enthalpy [kcal/mol]	Entropy [cal/mol/Kelvin]	Heat capacity [cal/mol/Kelvin]	Heat of formation [kcal/mol]
Phytic acid C_6_ H_18_ P_6_ O_24_	**-1,228,071 ± 63,141.3**	**36,340 ± 16,529.9**	**267,158 ± 47,749.6**	**169,495 ± 28,600.5**	**-1,453,309 ± 16,529.7**
CoQ10 C_59_ H_90_ O_4_	197,463 ± 96,848.4	70,613 ± 32,219.7	556,608 ± 91,694.3	329,468 ± 75,736	-146,475 ± 32,221.3
Deferoxamine C_25_ H_48_ N_6_ O_8_	-55,423 ± 71,481.3	42,692 ± 19,068.6	359,819 ± 54,422.4	195,289 ± 42,572.9	-310,199 ± 19,068.5

The best values are in bold, as illustrated in the text.

### Iron chelating potency of Chia and CoQ_10_

**Figure 2:**
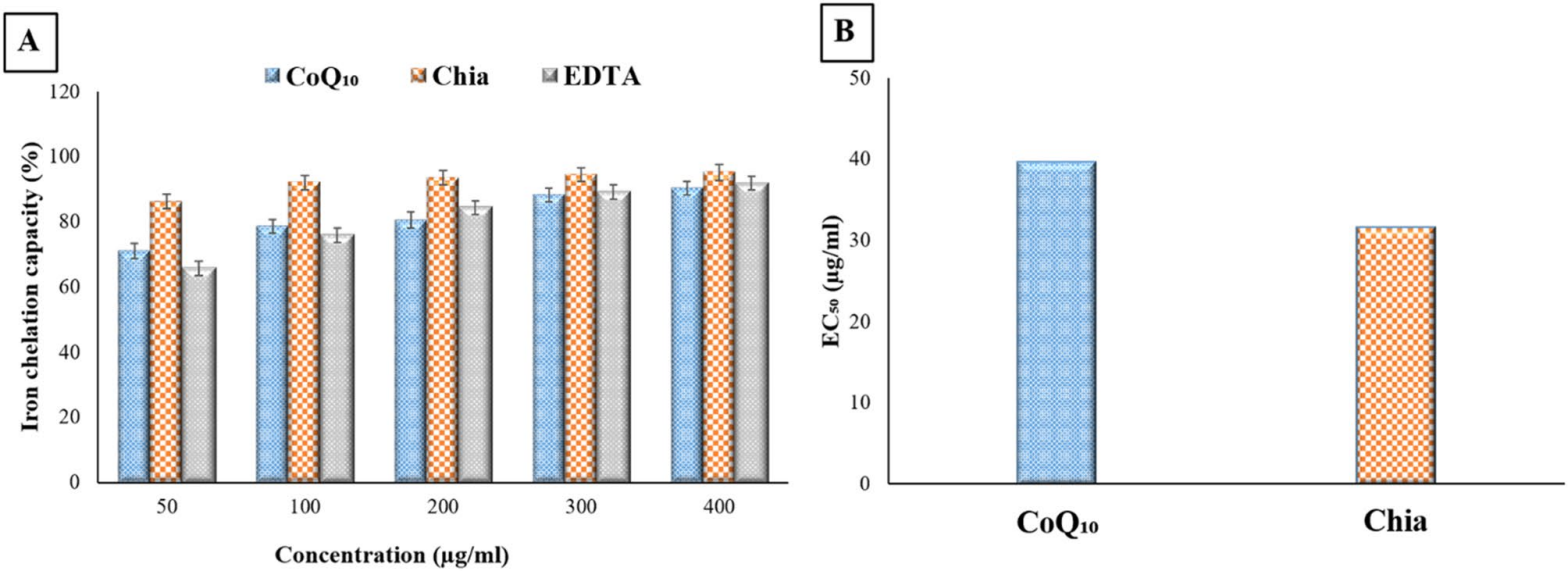
In vitro iron chelation potency of CoQ_10_ and Chia seeds. (**A**) Iron chelation capacity percentage of CoQ_10_ and Chia seeds. (**B**) Half-maximal effective concentration (EC_50_) *of the* CoQ_10_ and Chia seeds.

Figure 2A reveals that Chia and CoQ_10_ have a strong ion chelating potency as they could chelate iron to some extent comparable to EDTA (standard chelating agent). This is indicated by disrupting the formation of a violet colored Fe^2+^-ferrozine complex. Additionally, it was recorded that the iron chelating activity of both Chia and CoQ_10_ was positively correlated to their concentration. At a concentration of 50 µg/ml, the iron chelating potency of Chia was higher than that of CoQ_10_ and EDTA. As, Chia able to chelate more than 86.26% of the iron but CoQ_10_ and EDTA chelated only 71.08% and 65.72% of the iron, respectively at the same concentration. Attractively, Chia exhibited high iron chelating potency with a high percentage of 95.16% at a high concentration approximately as EDTA that chelated iron by 91.84% at the same concentration (Fig. 2A). Furthermore, the EC_50_ of Chia and CoQ_10_ were found to be 31.55 and 39.67 µg/ml, respectively suggesting that both Chia and CoQ_10_ could be utilized as a potent iron chelating agent (Fig. 2B).

### Effect of Chia and CoQ_10_ on food intake, body weight, and relative organs weight of iron overloaded mice

Table 4 demonstrates that IOL mice revealed marked (P < 0.05) increase in body weight and total food intake but this was statistically significant only in total food intake as compared to control mice. Meanwhile, the body weight and total food intake of IOL mice treated with Chia and CoQ_10_ significantly (P < 0.05) decreased when compared with the untreated IOL group. Conversely, the relative weights of the liver and kidney significantly (P < 0.05) increased in IOL mice, as compared to the control group. Interestingly, significant (P < 0.05) decline in the relative weights of the liver and kidney was noticed after therapy of Chia and CoQ_10_ in comparison with the untreated IOL group. Regarding the relative weight of the organs, a significant (P < 0.05) gain was recorded only in case of the relative kidney weight of mice after administration of Chia or CoQ_10_, as compared with control group. Also, dietary intake of Chia caused a significant (P < 0.05) loss in body weight; but oral administration of CoQ_10_ caused non-significant change in the body weight and total food intake as compared with control group.

**Table 4:** The modulating efficacies of dietary chia and CoQ_10_ on body weight change, relative organs weight and total food intake of iron-overloaded mice.

Experimental groups	Body weight change (g)	Relative liver weight (g/100 g body weight)	Relative kidney weight (g/100 g body weight)	Total food intake (g)
Control	6.69 ± 0.37^a^	5.30 ± 0.32^a^	1.15 ± 0.11^a^	408.00 ± 15.24^ab^
Chia	3.70 ± 0.58^b^	4.64 ± 0.19^a^	1.52 ± 0.01^b^	438.28 ± 11.33^b^
CoQ_10_	6.25 ± 0.31^ac^	5.06 ± 0.14^a^	1.38 ± 0.04^b^	400.71 ± 17.06^a^
IOL	7.46 ± 0.58^a^	7.77 ± 0.36^b^	1.79 ± 0.03^c^	477.14 ± 6.64^c^
IOL + Chia	2.38 ± 0.27^b^	5.33 ± 0.15^a^	1.20 ± 0.02^a^	450.00 ± 11.54^c^
IOL + CoQ_10_	2.61 ± 0.65^b^	4.91 ± 0.08^a^	1.48 ± 0.02^b^	431.57 ± 6.62^ab^
IOL + Chia + CoQ_10_	5.25 ± 0.43^c^	5.29 ± 0.14^a^	1.22 ± 0.02^a^	435.71 + 4.28^b^

Values are mean ± SEM (n = 7). Values with different superscript letters in the same raw are significantly different at P < 0.05.

COQ_10_: coenzyme Q_10_ (300 mg/kg body weight); Diet containing chia seeds (362 g/kg diet); IOL: Iron overload.

### Effect of Chia and CoQ_10_ on iron indices of iron overloaded mice

Iron-overloaded mice showed significant (P < 0.05) increase in iron, ferritin, transferrin, TIBC, and TSI as well as significant (P < 0.05) decrease in UIBC when compared with control mice (Table 5). Interestingly, IOL mice treated with Chia and/or CoQ_10_ for 4 weeks showed significant (P < 0.05) ameliorated undesirable changes in all above-mentioned iron indices when compared with the untreated IOL group. Dietary intake of Chia or administration of CoQ_10_ (300 mg/kg body weight) for 4 weeks caused non-significant change in the levels of serum iron, ferritin, and TSI, as compared to the control group. Furthermore, normal mice fed with Chia for 4 weeks showed a significant (P < 0.05) decline in TIBC and UIBC level, as compared to the control group. Meanwhile, a significant (P < 0.05) increase was recorded in the transferrin, TIBC and UIBC levels of mice administrated CoQ_10_ when compared with the control mice.

**Table 5:** The modulating efficacies of dietary chia and CoQ_10_ on serum iron indices of iron-overloaded mice.

Experimental groups	Iron (μg/dl)	Ferritin (ng/ml)	Transferrin (μg/dl)	TIBC (μg/dl)	UIBC (µg/dl)	TSI (%)
Control	53.45 ± 1.67^a^	0.15 ± 0.01^a^	449.61 ± 4.28^a^	519.52 ± 3.18^a^	466.07 ± 4.08^a^	10.29 ± 0.35^ab^
Chia	55.61 ± 1.94^a^	0.20 ± 0.03^ab^	444.66 ± 2.10^a^	507.66 ± 0.67^c^	452.05 ± 1.98^c^	10.95 ± 0.38^b^
CoQ_10_	54.65 ± 0.54^a^	0.21 ± 0.01^ab^	456.95 ± 2.62^b^	542.48 ± 1.24^b^	487.82 ± 1.21^b^	10.10 ± 0.09^a^
IOL	121.42 ± 1.06^b^	0.29 ± 0.01^c^	474.85 ± 1.18^c^	552.90 ± 1.93^d^	431.48 ± 2.34^d^	21.96 ± 0.57^c^
IOL + Chia	55.28 ± 0.29^a^	0.23 ± 0.01^b^	466.64 ± 1.16^d^	502.00 ± 0.89^e^	446.71 ± 0.95^c^	11.01 ± 0.06^d^
IOL + CoQ_10_	55.20 ± 0.28^a^	0.20 ± 0.01^ab^	467.86 ± 1.40^d^	504.33 ± 1.57^c^	449.13 ± 2.71^c^	10.94 ± 0.05^bd^
IOL + Chia + CoQ_10_	52.75 ± 0.56^a^	0.17 ± 0.01^ab^	462.62 ± 0.58^bd^	497.64 ± 1.62^e^	444.88 ± 1.99^e^	10.60 ± 0.05^bd^

Values are mean ± SEM (n = 7). Values with different superscript letters in the same raw are significantly different at P < 0.05.

CoQ_10_: Coenzyme Q_10_ (300 mg/kg body weight); Diet containing Chia seeds (362 g/kg diet); IOL: Iron overload.

### Effect of Chia and CoQ_10_ on hematological indices of iron overloaded mice

Iron-overloaded mice showed significant (P < 0.05) elevation in RBCs, Hb, HCT, MCV, MCH indices and WBCs but significant (P < 0.05) decline in their platelets count was noticed when compared to control mice (Tables 6, 7). Meanwhile, mice administrated either Chia or CoQ_10_ (300 mg/kg body weight) for 4 weeks showed a non-significant change in RBCs, HCT, MCV, WBCs and platelets as compared to control mice (Tables 6, 7). On the other hand, dietary intake of Chia caused a significant (P < 0.05) increment in the Hb content of normal mice as compared to the control group. Meanwhile, the MCH level of CoQ_10_ group decreased significantly (P < 0.05) when compared with the control group.

**Table 6:** The modulating efficacies of dietary chia and CoQ_10_ on RBCs indices of iron-overloaded mice.

Experimental groups	Hb (g/dl)	RBCs (million/cm)	HCT (%)	MCV (fl/cell)	MCH (pg/cell)
Control	12.16 ± 0.34^ab^	8.17 ± 0.10^a^	41.71 ± 0.49^ab^	50.96 ± 1.41^ab^	15.38 ± 0.18^a^
Chia	13.57 ± 0.25^c^	8.48 ± 0.04^a^	43.68 ± 2.12^b^	53.03 ± 0.92^ab^	15.72 ± 0.12^ac^
CoQ_10_	12.25 ± 0.47^ab^	8.15 ± 0.35^a^	43.38 ± 1.83^b^	54.10 ± 0.50^b^	14.96 ± 0.11^b^
IOL	14.41 ± 0.11^d^	9.33 ± 0.05^b^	49.81 ± 0.61^c^	59.62 ± 0.46^c^	17.09 ± 0.12^d^
IOL + Chia	12.67 ± 0.14^b^	7.51 ± 0.05^cd^	38.96 ± 0.35^ad^	52.62 ± 0.16^ab^	16.40 ± 0.09^e^
IOL + CoQ_10_	12.00 ± 0.11^ab^	7.57 ± 0.03^c^	38.54 ± 1.33^ad^	51.96 ± 0.89^ab^	15.86 ± 0.18^c^
IOL + Chia + CoQ_10_	11.86 ± 0.02^a^	7.11 ± 0.12^d^	36.37 ± 0.12^d^	48.57 ± 0.47^d^	16.26 ± 0.08^e^

Values are mean ± SEM (n = 7). Values with different superscript letters in the same raw are significantly different at P < 0.05.

CoQ_10_: Coenzyme Q_10_ (300 mg/kg body weight); Diet containing Chia seeds (362 g/kg diet); IOL: Iron overload.

**Table 7:** The modulating efficacies of dietary chia and CoQ_10_ on WBCs and platelets of iron-overloaded mice.

Experimental groups	WBCs (10^3^/mm^3^)	Platelets (10^3^/mm^3^)
Control	7.76 ± 0.56^a^	1608.57 ± 41.33^a^
Chia	7.37 ± 0.37^ab^	1440.36 ± 30.83^ab^
CoQ_10_	7.69 ± 0.70^a^	1603.05 ± 111.36^a^
IOL	10.06 ± 0.43^c^	1177.57 ± 50.07^c^
IOL + Chia	6.74 ± 0.10^bd^	1197.14 ± 52.27^c^
IOL + CoQ_10_	5.26 ± 0.76^d^	1333.33 ± 66.52^cb^
IOL + Chia + CoQ_10_	5.91 ± 0.47^bd^	1453.64 ± 33.84^ab^

Values are mean ± SEM (n = 7). Values with different superscript letters in the same raw are significantly different at P < 0.05.

CoQ_10_: Coenzyme Q_10_ (300 mg/kg body weight); Diet containing Chia seeds (362 g/kg diet); IOL: Iron overload.

### Effect of Chia and CoQ_10_ on hepatic and renal markers of iron overloaded mice

Marked hepatorenal failure was observed in IOL mice and this confirmed by significant (P < 0.05) elevation in hepatorenal function markers (AST, ALT, and creatinine), as compared with the control group (Table 8). Meanwhile, total protein and albumin content significantly decreased after IPC administration respecting to control group. Notably, treatment with Chia and CoQ_10_ for 4 weeks caused significant (P < 0.05) improvement in all aforementioned hepatorenal function markers, as compared to untreated IOL mice (Table 8). Mice administrated Chia or CoQ_10_ showed no undesirable effect on hepatorenal function markers, as compared to control mice.

**Table 8:** The modulating efficacies of dietary chia and CoQ_10_ on hepatic and renal function markers of iron-overloaded mice.

Experimental groups	ALT (U/L)	AST (U/L)	Total protein (g/dl)	Albumin (g/dl)	Creatinine (mg/dl)
Control	27.01 ± 0.55^a^	35.71 ± 3.78^a^	28.19 ± 2.02^a^	1.88 ± 0.05^a^	0.86 ± 0.02^a^
Chia	19.99 ± 1.43^b^	38.08 ± 4.34^a^	26.65 ± 1.63^a^	2.45 ± 0.02^c^	0.78 ± 0.01^b^
CoQ_10_	28.28 ± 1.07^a^	10.47 ± 0.98^b^	28.82 ± 1.72^a^	2.00 ± 0.06^ab^	0.86 ± 0.02^a^
IOL	55.26 ± 4.56^c^	85.36 ± 2.71^c^	21.48 ± 1.22^b^	0.92 ± 0.04^d^	1.27 ± 0.03^c^
IOL + Chia	22.86 ± 1.01^ab^	40.47 ± 5.87^a^	25.90 ± 0.16^a^	1.94 ± 0.02^ab^	0.67 ± 0.03^d^
IOL + CoQ_10_	22.05 ± 0.41^ab^	54.00 ± 0.91^e^	35.29 ± 0.14^c^	2.04 ± 0.05^be^	0.90 ± 0.03^a^
IOL + Chia + CoQ_10_	19.45 ± 2.31^b^	24.99 ± 0.37^b^	54.05 ± 1.90^d^	2.13 ± 0.03^e^	0.64 ± 0.02^d^

Values are mean ± SEM (n = 7). Values with different superscript letters in the same raw are significantly different at P < 0.05.

CoQ_10_: Coenzyme Q_10_ (300 mg/kg body weight); Diet containing Chia seeds (362 g/kg diet); IOL: Iron overload.

### Effect of Chia and CoQ_10_ on oxidative/antioxidative markers of iron overloaded mice

Iron-overloaded mice showed an imbalance between oxidative and antioxidant markers inducing oxidative stress status in hepatic and renal tissues. This is confirmed by significant (P < 0.05) increment in MDA content as well as significant (P < 0.05) decline in GSH, SOD, and GR levels in both hepatic and renal tissues, as compared to the control mice. Meanwhile, IOL mice revealed a significant (P < 0.05) decline in their CAT activity in renal tissue only in comparison with the control group. Attractively, significant (P < 0.05) amelioration in MDA, GSH, SOD, CAT, and GR concentrations was recorded in IOL mice treated with Chia and CoQ_10_, as compared with IOL mice. Dietary intake of Chia or oral administration of CoQ_10_ has no deleterious effect on most of the oxidative and antioxidant markers in hepatorenal tissues, as compared to the control group (Tables 9, 10).

**Table 9:** The modulating efficacies of dietary chia and CoQ_10_ on hepatic oxidative/antioxidative markers of the iron-overloaded mice.

Experimental groups	Hepatic MDA (nmole/g protein)	Hepatic GSH (mg/g protein)	Hepatic SOD (U/g protein)	Hepatic CAT (U/g protein)	Hepatic GR (U/g protein)
Control	57.99 ± 0.75^a^	13.26 ± 0.63^a^	160.62 ± 8.96^a^	332.48 ± 39.92^a^	78.68 ± 4.62^a^
Chia	50.07 ± 1.30^c^	10.83 ± 0.26^b^	127.90 ± 13.65^b^	358.95 ± 14.54^a^	66.93 ± 4.10^b^
CoQ_10_	55.89 ± 0.71^ab^	11.43 ± 0.97^b^	161.93 ± 4.44^a^	361.52 ± 15.90^a^	73.01 ± 0.71^abd^
IOL	68.86 ± 2.87^d^	5.17 ± 0.33^c^	103.88 ± 3.94^c^	181.23 ± 27.60^a^	54.69 ± 1.89^c^
IOL + Chia	57.83 ± 0.48^a^	6.90 ± 0.33^d^	130.92 ± 6.66^b^	894.47 ± 49.56^b^	79.01 ± 3.40^ad^
IOL + CoQ_10_	51.25 ± 2.19^bc^	5.83 ± 0.11^cd^	130.65 ± 2.66^b^	1405.68 ± 78.25^c^	70.55 ± 4.12^ab^
IOL + Chia + CoQ_10_	49.99 ± 2.78^c^	14.03 ± 0.05^a^	140.32 ± 6.13^ab^	1036.88 ± 17.15^b^	83.81 ± 4.30^d^

Values are mean ± SEM (n = 7). Values with different superscript letters in the same raw are significantly different at P < 0.05.

CoQ_10_: Coenzyme Q_10_ (300 mg/kg body weight); Diet containing Chia seeds (362 g/kg diet); IOL: Iron overload.

**Table 10:** The modulating efficacies of dietary chia and CoQ_10_ on renal oxidative/antioxidative markers of the iron-overloaded mice.

Experimental groups	Renal MDA (nmole/g protein)	Renal GSH (mg/g protein)	Renal SOD (U/g protein)	Renal CAT (U/g protein)	Renal GR (U/g protein)
Control	48.19 ± 2.38^a^	20.77 ± 2.54^a^	566.64 ± 5.45^a^	953.52 ± 95.56^a^	33.12 ± 0.56^a^
Chia	48.55 ± 1.97^a^	17.81 ± 1.15^ac^	349.93 ± 39.60^c^	734.56 ± 87.16^b^	29.00 ± 4.15^a^
CoQ_10_	44.01 ± 2.20^a^	13.31 ± 0.70^bd^	425.03 ± 42.98^b^	1110.01 ± 119.42^a^	31.34 ± 2.73^a^
IOL	63.00 ± 0.66^b^	10.07 ± 0.81^d^	65.04 ± 4.35^d^	379.37 ± 25.30^c^	17.23 ± 2.34^b^
IOL + Chia	49.99 ± 3.19^ac^	14.69 ± 0.17^bc^	76.67 ± 2.82^d^	523.07 ± 26.71^bd^	26.34 ± 2.17^a^
IOL + CoQ_10_	54.99 ± 0.61^c^	14.01 ± 1.14^bc^	78.54 ± 2.19^d^	376.96 ± 27.14^cd^	24.88 ± 3.55^a^
IOL + Chia + CoQ_10_	45.53 ± 1.10^a^	17.04 ± 1.16^abc^	205.40 ± 19.41^e^	695.03 ± 61.85^b^	28.81 ± 0.38^a^

Values are mean ± SEM (n = 7). Values with different superscript letters in the same raw are significantly different at P < 0.05.

CoQ_10_: Coenzyme Q_10_ (300 mg/kg body weight); Diet containing Chia seeds (362 g/kg diet); IOL: Iron overload.

### Effect of Chia and CoQ_10_ on mitochondrial membrane potential change (Δψ) of iron overloaded mice

**Figure 3:**
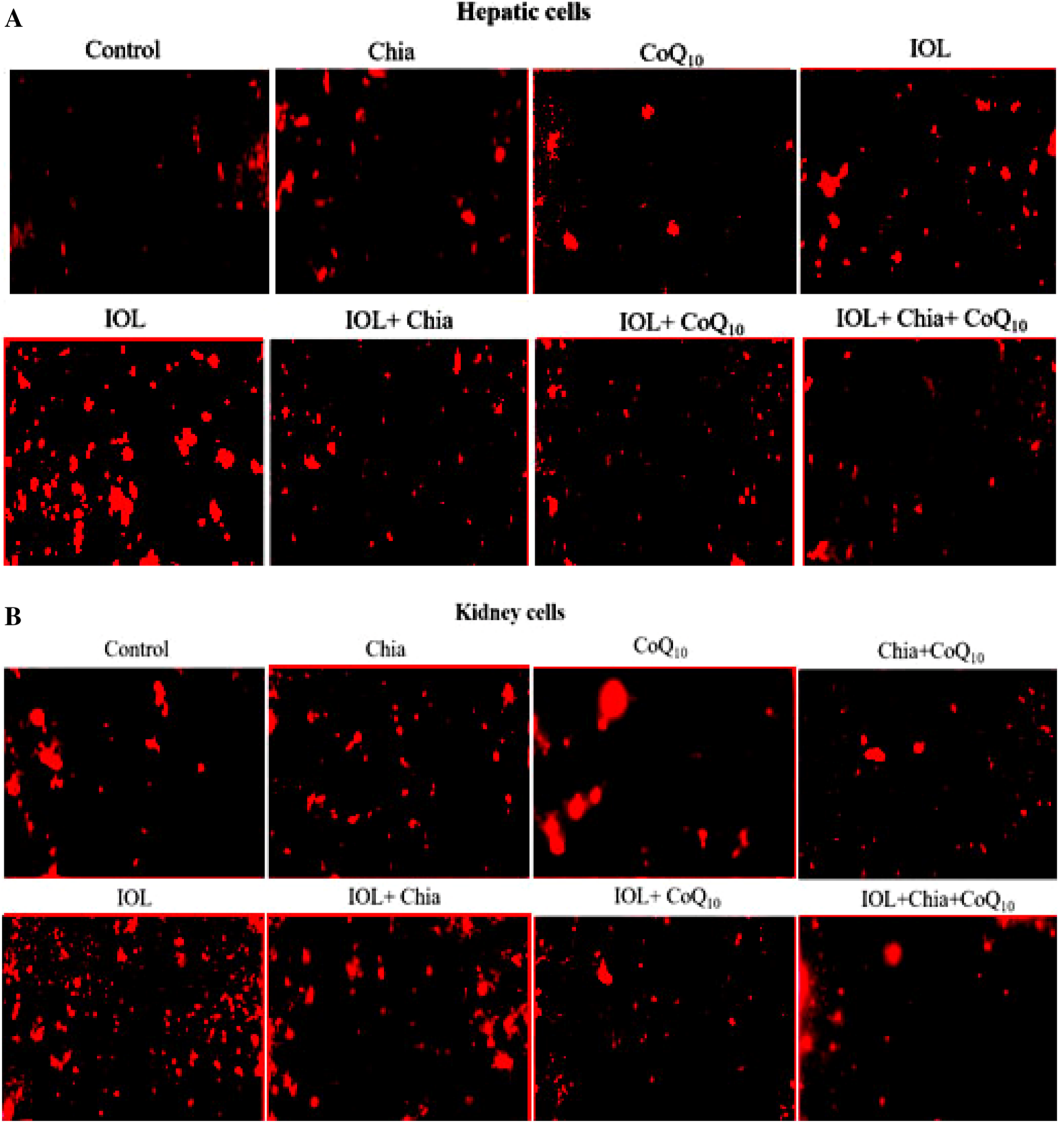
(**A**) The modulating efficacies of dietary chia and CoQ_10_ on the hepatic mitochondrial membrane potential (Δψ) of iron-overloaded mice. CoQ_10_: Coenzyme Q_10_ (300 mg/kg body weight); Diet containing Chia seeds (362 g/kg diet); IOL: Iron overload. (**B**) The modulating efficacies of dietary chia and CoQ_10_ on the renal mitochondrial membrane potential (Δψ) of iron-overloaded mice. CoQ_10_: Coenzyme Q_10_ (300 mg/kg body weight); Diet containing Chia seeds (362 g/kg diet); IOL: Iron overload.

As shown in Fig. 3A,B, iron overload caused Δψ depolarization as indicated by remarkably decreased brightness of fluorescence emitted from hepatic and renal cells stained with Rhodamine 123 dye comparable to that of the control cells. On the other hand, monotherapy with Chia or CoQ_10_ or slightly restored the hepatic and renal mitochondrial depolarization, as compared to the untreated IOL group. Attractively, the combined treatment (Chia + CoQ_10_) caused the most pronounced amelioration in mitochondrial membrane potential as confirmed by extraordinarily increased brightness of fluorescence emitted from hepatic and renal cells respecting to that of the untreated IOL group. Dietary intake of Chia and CoQ_10_ administration and have no undesirable effect on Δψ of both hepatic and renal mitochondria when compared with the control group. This is confirmed by the observed non-changes in the brightness of fluorescence emitted from the liver and renal cells stained with Rhodamine 123 dye.

### Effect of Chia and CoQ_10_ on DNA fragmentation of iron overloaded mice

The degree of DNA fragmentation in hepatic and renal tissues was more obvious in IOL mice than in the control mice and this is confirmed by a significant (P < 0.05) increase in the % fragmented DNA (Table 11). Interestingly, the degree of DNA damage significantly declined in hepatic and renal tissues of IOL mice treated either with Chia or CoQ_10_ (300 mg/kg b.wt) or for 4 weeks, as compared to untreated IOL group. Furthermore, IOL mice treated with the combined Chia and CoQ_10_ for 4 weeks demonstrated the most amelioration in the % fragmented DNA for hepatic and renal tissues with percentage of improvement of 37.52% and 34.90%, respectively, comparable to that of the untreated IOL group. Mice administered Chia or CoQ_10_ or for 4 weeks showed no marked detrimental effect on DNA integrity in both hepatic and renal tissue, as compared to control mice.

**Table 11:** Modulating efficacies of dietary chia and CoQ_10_ on DNA fragmentation of iron-overloaded mice.

Experimental groups	Hepatic fragmented DNA %	Renal fragmented DNA %
Control	48.41 ± 0.27^a^	47.35 ± 0.23^a^
Chia	44.91 ± 0.39^c^	47.86 ± 0.46^a^
CoQ_10_	47.86 ± 0.26^ab^	47.83 ± 0.31^a^
IOL	74.39 ± 0.67^d^	68.47 ± 0.75^b^
IOL + Chia	50.18 ± 0.22^e^	51.42 ± 0.78^d^
IOL + CoQ_10_	50.18 ± 0.35^e^	48.23 ± 0.52^a^
IOL + Chia + CoQ_10_	46.48 ± 0.89^b^	44.57 ± 0.26^c^

Values are mean ± SEM (n = 7). Values with different superscript letters in the same raw are significantly different at P < 0.05.

CoQ_10_: Coenzyme Q_10_ (300 mg/kg body weight); Diet containing chia seeds (362 g/kg diet); IOL: Iron overload.

### Effect of Chia and CoQ_10_ on mitochondrial DNA copies of iron over-loaded mice

**Figure 4:**
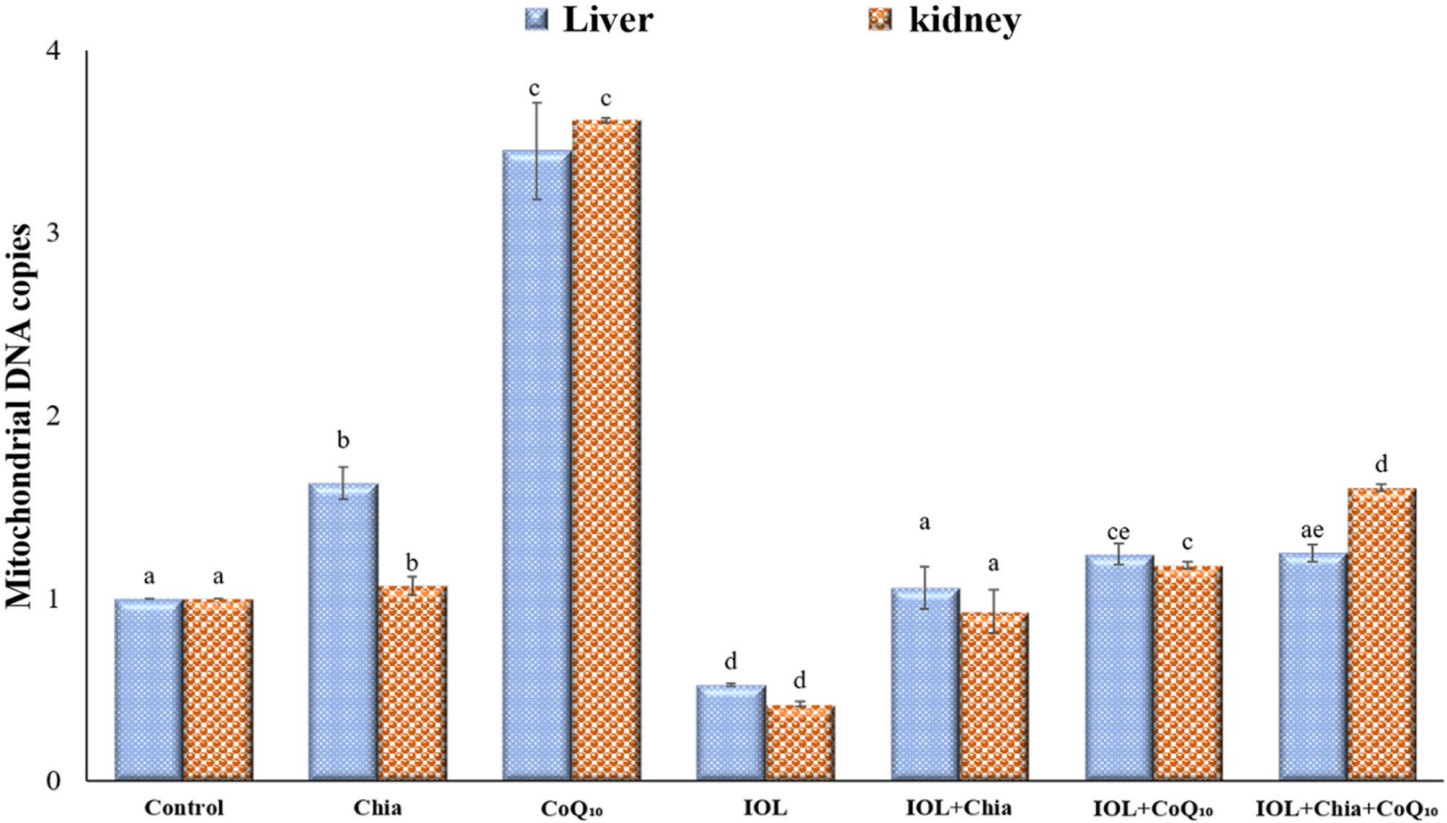
Modulating efficacies of dietary chia and CoQ_10_ on mitochondrial DNA copies of iron-overloaded mice. Values are mean ± SEM (n = 7). Values with different superscript letters in the same raw are significantly different at P < 0.05. CoQ_10_: Coenzyme Q_10_ (300 mg/kg body weight); Diet containing Chia seeds (362 g/kg diet); IOL: Iron overload.

The number of mitochondrial DNA copies was significantly suppressed in hepatic and renal tissues of IOL mice when compared with the control mice (Fig. 4). On the other hand, treatment with Chia and CoQ_10_ either separately or combined caused a marked increase in the number of mitochondrial DNA copies in hepatic and renal tissues when compared with untreated IOL group. Also, Fig. 4 reveals that dietary intake of Chia or oral administration of CoQ_10_ (300 mg/kg b.wt) or for 4 weeks caused a significant (P < 0.05) increase in the number of mitochondrial DNA copies in hepatic and renal tissues, as compared to the control group.

### Effect of Chia and CoQ_10_ on histological changes of iron over-loaded mice

**Figure 5:**
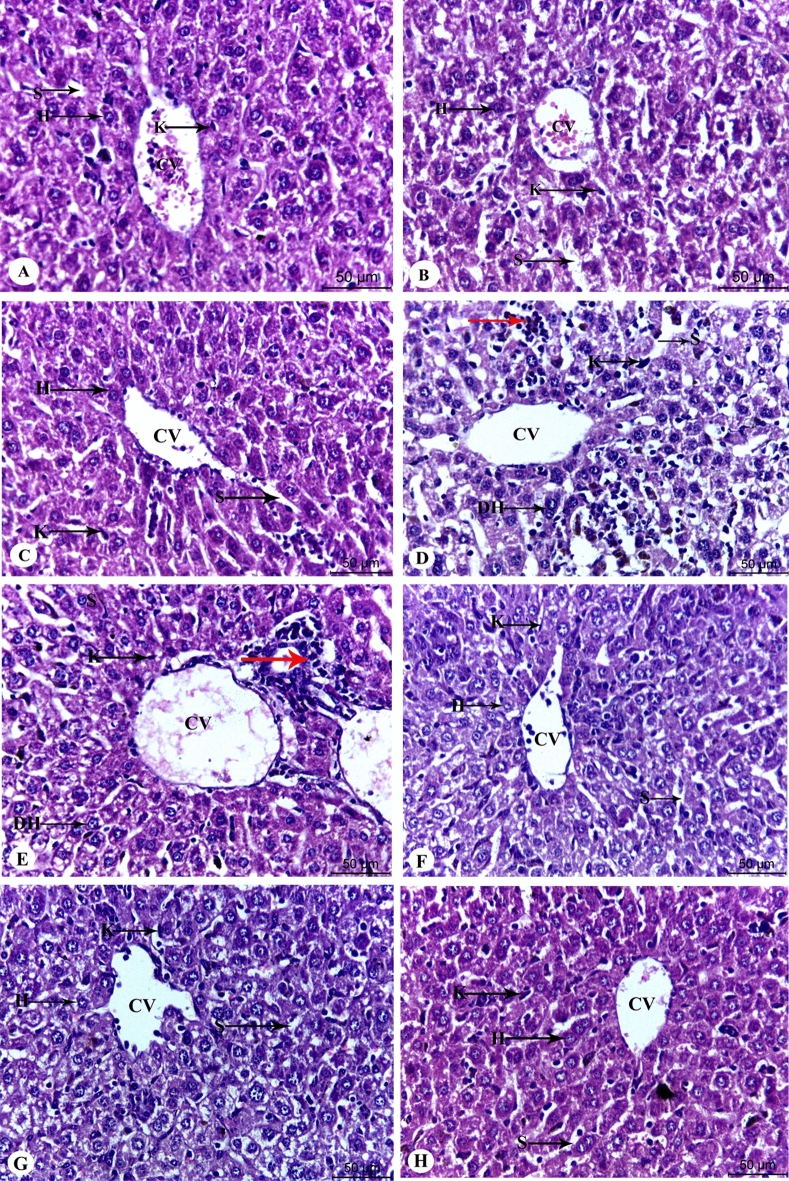
Photomicrographs of the mice's liver architecture of different experimental groups stained with hematoxylin and eosin. (**A**) control group showing well-preserved liver architecture with round polygonal hepatocytes, normal distribution of sinusoidal spaces and Kupffer cells. (**B**,**C**) Dietary Chia and CoQ_10_ liver sections demonstrate distinct and relatively normal hepatic architecture. (**D**,**E**) The iron overload group showed severe hepatic injury with inflammatory infiltration in the sinusoidal spaces, enlarged kupffer cells and degenerated hepatocytes. (**F**,**G**) Iron-overloaded mice treated with dietary Chia or CoQ_10_ showed slight amelioration in hepatic structure with few degenerated hepatocytes. (H) Iron-overloaded mice treated with combined therapy (Chia and CoQ_10_) showed more pronounced improvement of the liver structure, confirmed by normal sinusoids and central vein. H: Hepatocyte, S: Sinusoidal space, K: Kupffer cell, CV: Central vein, DH: Degenerated hepatocyte. The red arrow refers to inflammatory infiltration.

Concerning hepatic architecture, Fig. 5 A demonstrates regular histology of hepatic tissue which displayed the well-organized lobular architecture with hepatocyte (H) arranged in cords and regularly aligned sinusoids (S) in the control group. Also, the hepatic architecture of Chia or CoQ_10_ mice revealed more or less normal hepatic structure with slight inflammatory cells (Fig. 5B,C). Conversely, IOL caused hepatic damage as confirmed by substantial changes in hepatic tissue including hepatocellular necrosis and apoptosis, mononuclear cell infiltration, diffused Kupffer cells, and hyperplasia compared to the healthy control group (Fig. 5D,E). Interestingly, IOL mice treated with Chia and CoQ_10_ improved the liver structure, confirmed by normal sinusoids and central veins (Fig. 5F–H).

**Figure 6:**
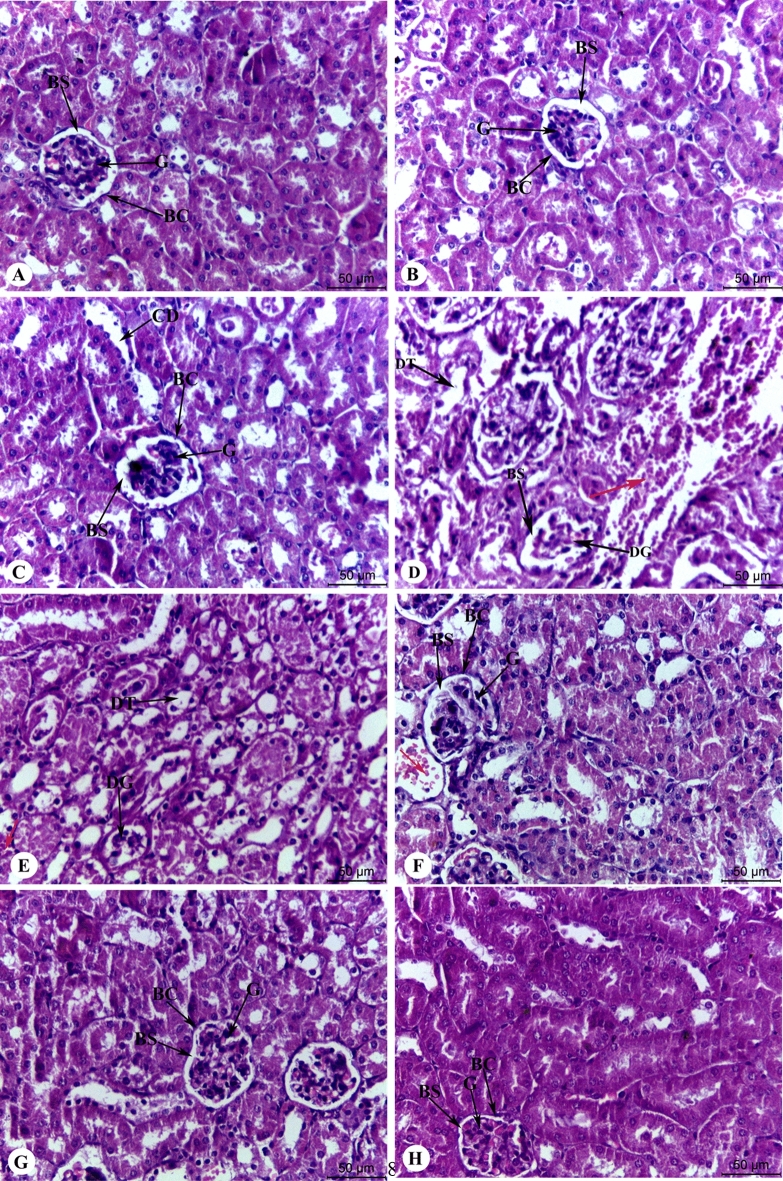
Photomicrographs of the mice's kidney architecture of different experimental groups stained with hematoxylin and eosin. (**A**) The control group showed normal renal structure with renal corpuscles surrounded by Bowman’s capsule and intact proximal and distal convoluted tubules. (**B**,**C**) Dietary Chia and CoQ_10_ kidney sections show distinct and relatively normal kidney architecture. (**D**,**E**) The iron overload group showed severe renal injury with glomerular degeneration (DG), glomerular atrophy, tubular degeneration and lymphocyte distribution (red arrow). (**F**,**G**) Iron-overloaded mice treated with dietary Chia and CoQ_10_ showed more or less normal appearance of the control group with slight lymphocyte distribution (red arrow). (**H**) Iron-overloaded mice treated with combined dietary Chia and CoQ_10_ showed a marked recovery of the renal structure, as indicated by neither destruction of tubular structure nor degenerated proximal tubule. G: glomeruli, BS: Bowman’s space, BC: Bowman’s capsule, CD: collecting duct, DT: degenerated tubule.

Regarding the renal structure, renal sections of control, Chia, and CoQ_10_ mice showed typical renal architecture with renal corpuscles enclosed by Bowman’s capsule, and intact proximal and distal convoluted tubules (Fig. 6A–C). Meanwhile, excess IOL caused severe morphological alterations in the renal structure, including glomerular degeneration, glomerular basement membrane thickening, tubular degeneration, distribution of lymphocytes, and dilatation in Bowman’s space (Fig. 6D,E). Interestingly, IOL mice treated with Chia and CoQ_10_ caused marked amelioration in renal architecture, as signified by neither glomerular damage nor degenerated tubules, as compared with untreated IOL mice (Fig. 6F–H).

### Percentage improvement of Chia and CoQ_10_

**Figure 7:**
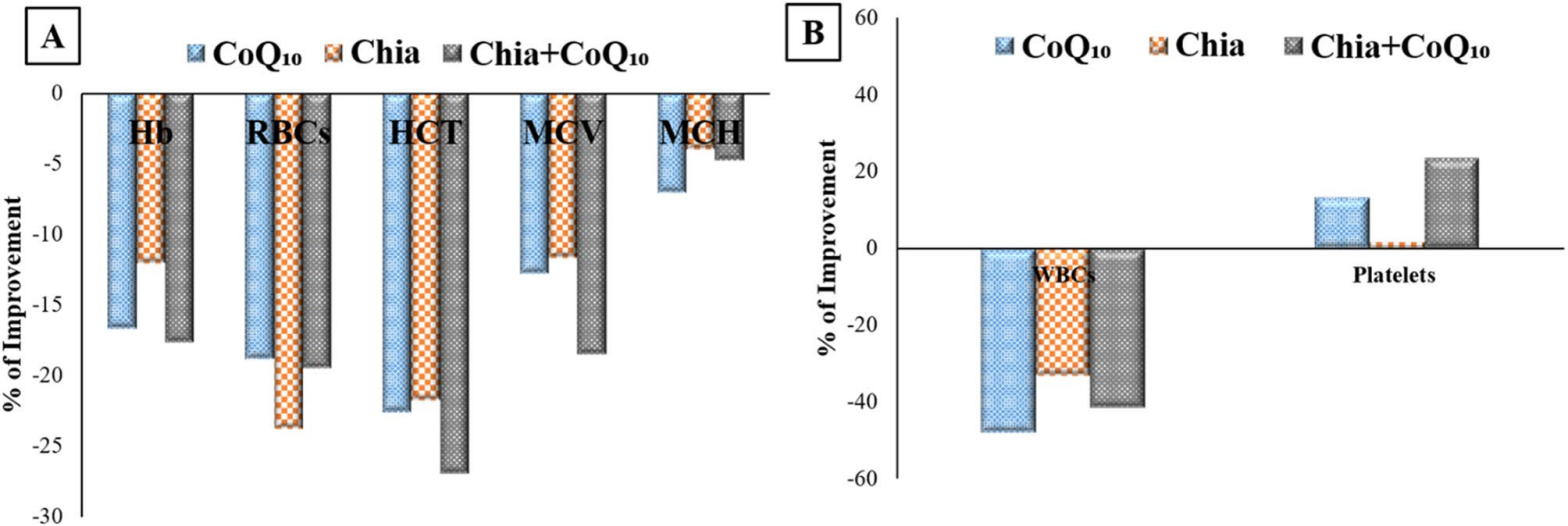
Improvement percentage of monotherapy (Chia or CoQ_10_) and combined therapy (Chia + CoQ_10_) on hematological parameters.

**Figure 8:**
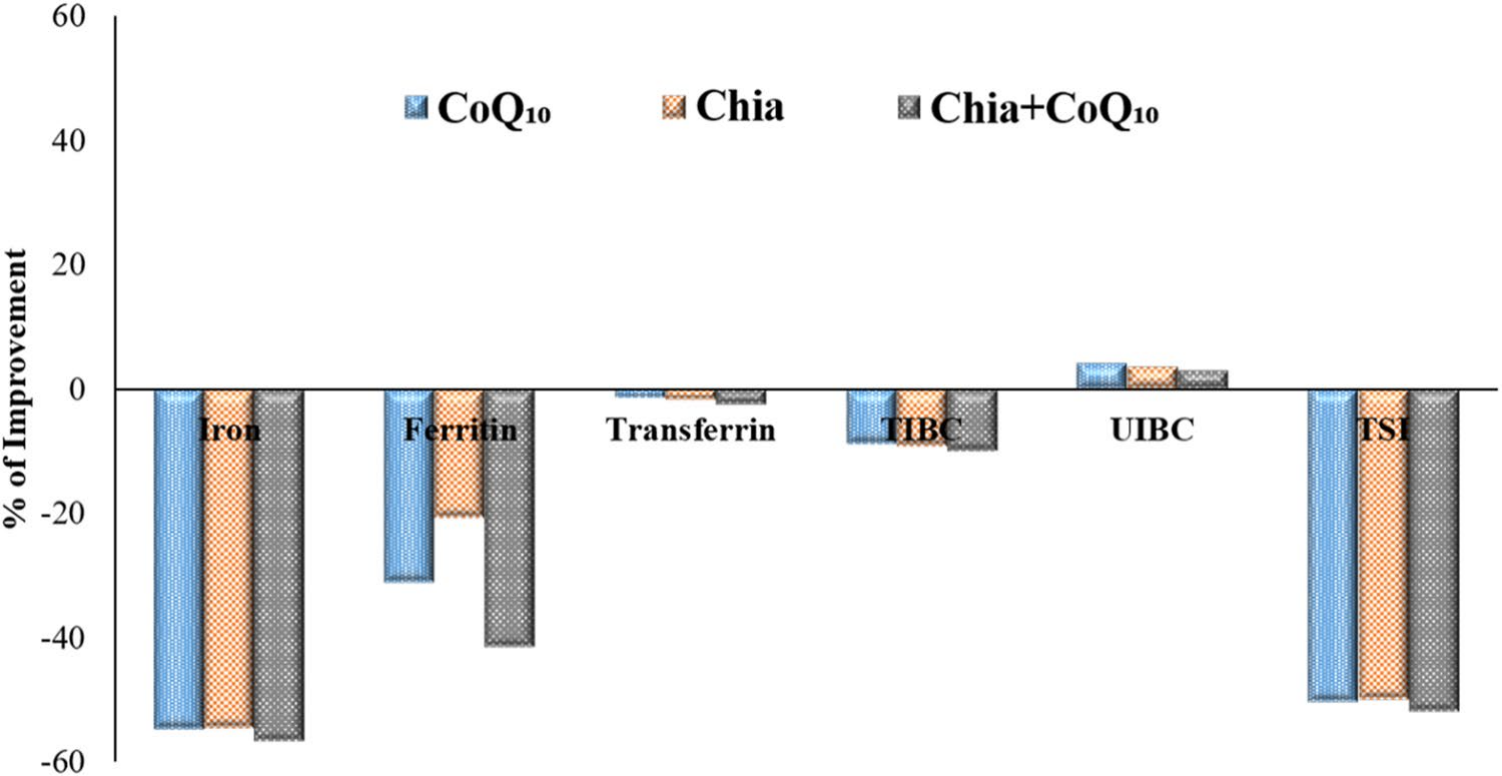
Improvement percentage of monotherapy (Chia or CoQ_10_) and combined therapy (Chia + CoQ_10_) on iron indices.

**Figure 9:**
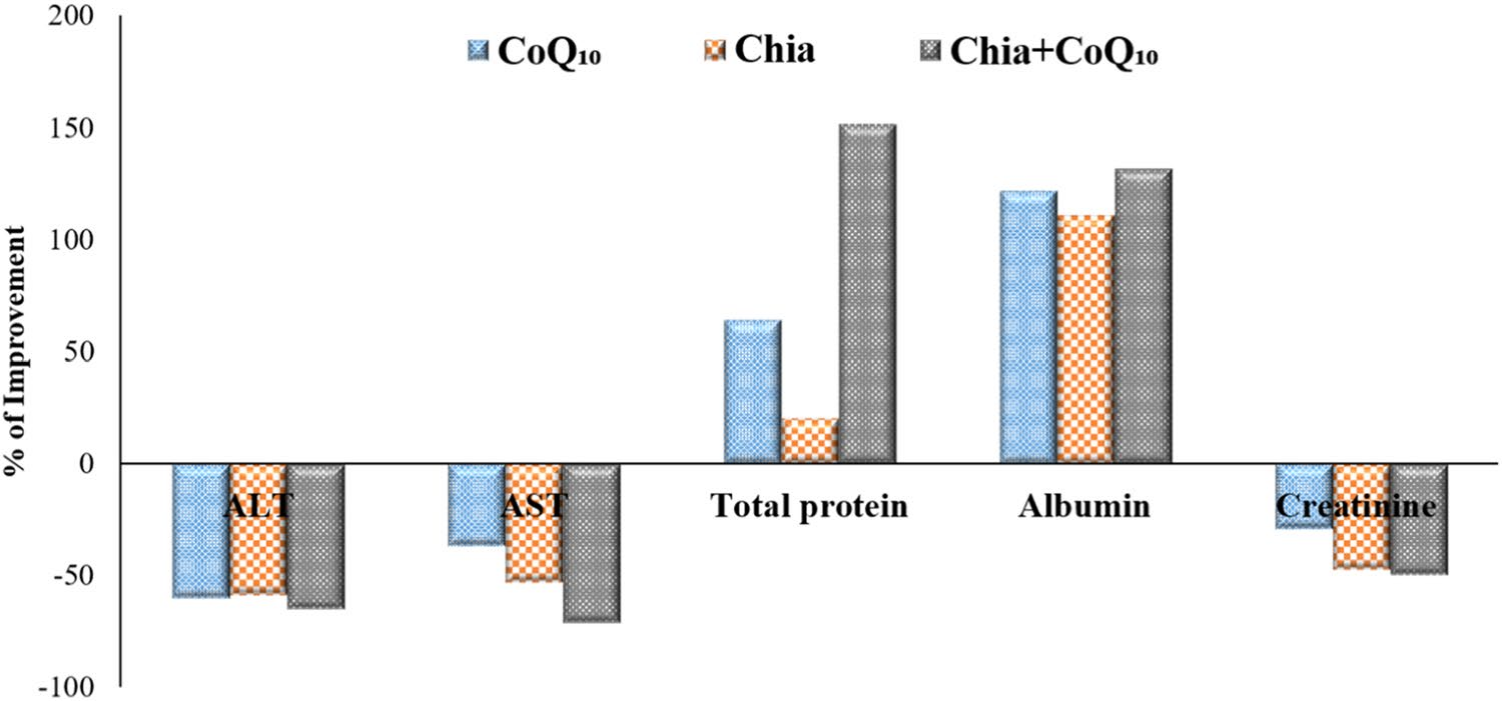
Improvement percentage of monotherapy (Chia or CoQ_10_) and combined therapy (Chia + CoQ_10_) on hepatorenal function markers.

**Figure 10:**
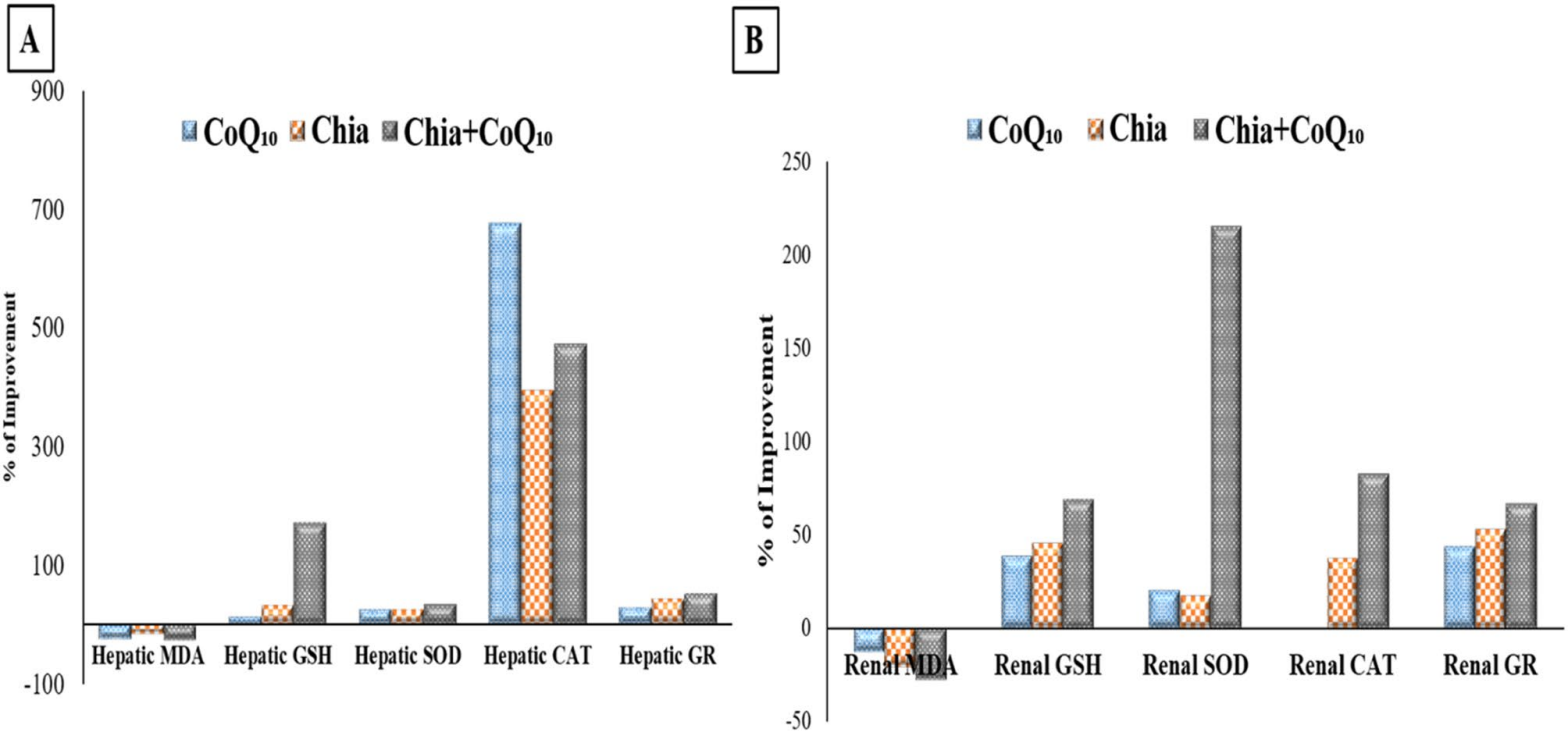
Improvement percentage of monotherapy (Chia or CoQ_10_) and combined therapy (Chia + CoQ_10_) on oxidative/antioxidative stress markers.

**Figure 11:**
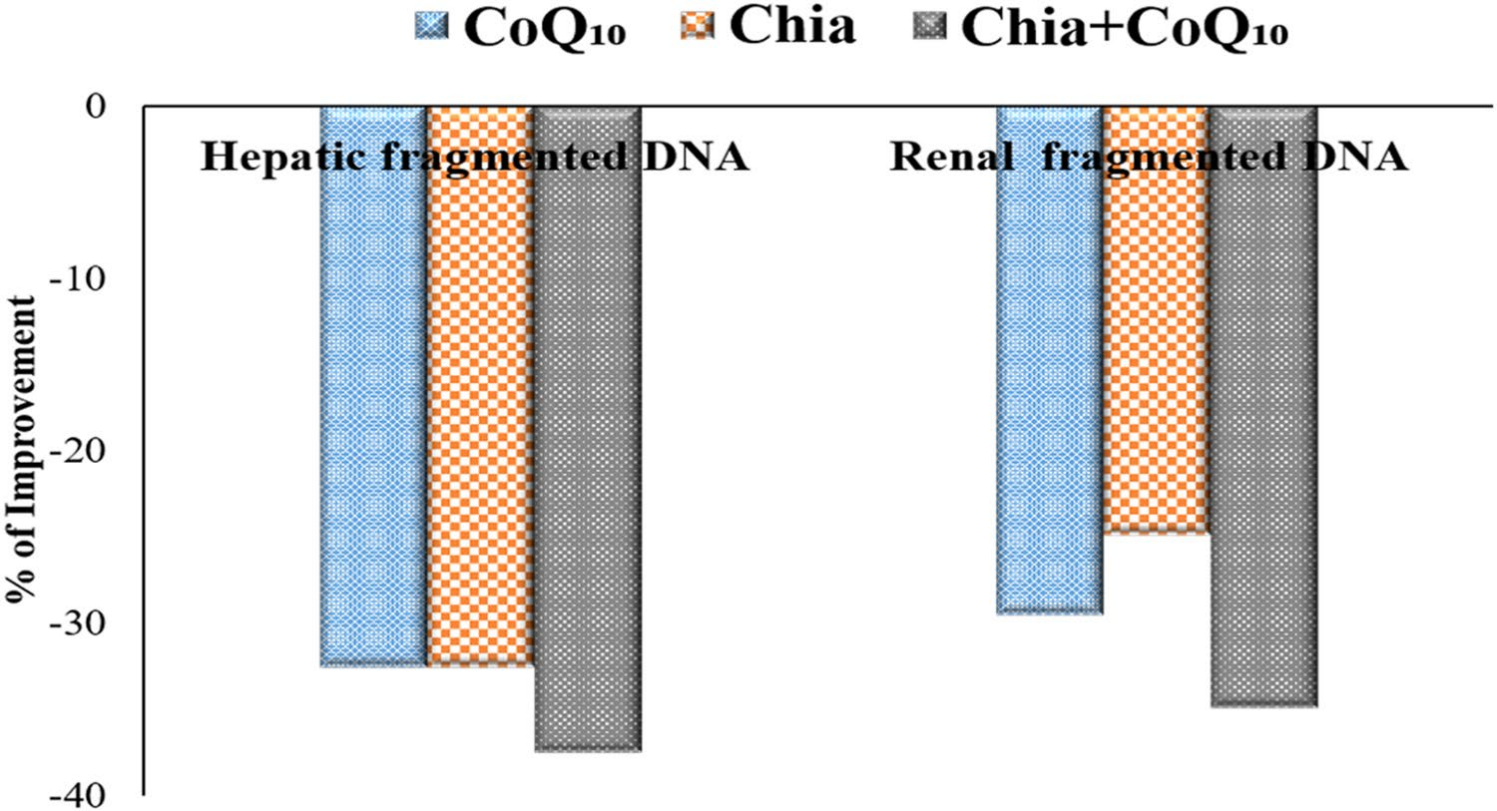
Improvement percentage of monotherapy (Chia or CoQ_10_) and combined therapy (Chia + CoQ_10_) on DNA fragmentation.

The findings, as mentioned earlier demonstrated that both Chia and CoQ_10_ were able to recover, to some extent, the altered estimated parameters induced by iron toxicity. Whereas, it has seemed that the combination of Chia and CoQ_10_ treatment is more effective than monotherapy because it can improve most of the altered hematological parameters, especially Hb, HCT, MCV, and platelets rather than monotherapy with an improvement percentage of 17.69%, 26.98%, 18.53%, and 23.44%, respectively *versus* the untreated IOL group (Fig. 7A,B). Also, the combined treatment (Chia + CoQ_10_) caused a marked amelioration in all iron indices, especially iron, ferritin, and TSI with a percentage of improvement 56.55%, 41.37%, 51.73%, respectively in comparison with the untreated IOL group (Fig. 8). Interestingly, combined treatment can improve the hepatorenal functions including AST, ALT, total protein, albumin and creatinine of IOL mice to 70.65%, 64.8%, 151.63%, 131.52, 49.6%, respectively rather than monotherapy *versus* the untreated IOL group (Fig. 9). Notably, combined treatment has more prominent ability than monotherapy to improve the abnormal alteration in hepatic oxidative/antioxidative stress markers (MDA, GSH, SOD, and GR) to 27.40%, 171.37%, 35.07%, 53.24% respectively. Similarly, renal oxidative/antioxidative stress markers (MDA, GSH, SOD, CAT, and GR) improved by 27.73%, 69.21%, 215.80%, 83.20%, 67.21%, respectively than the IOL group (Fig. 10A,B). Regarding the percentage of fragmented DNA, Chia + CoQ_10_ has a more pronounced efficiency in alleviating hepatic and renal DNA fragmentation, with a percentage of improvement of 37.52% and 37.90%, respectively (Fig. 11).

## Discussion

Iron, a potential partner in redox reactions, plays a big role in the biological system due to its reactive nature and lack of an excretory pathway^36^. Iron accumulation induced organ damage and toxicity by generating free radicals promoting lipid peroxidation^37^. Nowadays, there is great attention to utilizing natural phytochelator resources and antioxidant agents as promising co-adjuvants in the pharmaceutical therapy of IOL^38,39^. Therefore, the present study evaluated the prospective efficacy of Chia seeds, a potent phytochelator, and CoQ_10_, a potent antioxidant, separately or combined on IOL induced by ferric hydroxide polymaltose complex (IPC) in mice.

Iron overload is achieved in the present study by excessive iron deposition in the blood after IPC administration, promoting hepatocyte and renal damage. Additionally, the increased iron level in the IOL mice was associated with increased ferritin, transferrin, TIBC, and TSI% levels and decreased serum UIBC. Such changes resulted in the elevation of labile-free non-transferrin binding iron (NTBI) in the blood due to saturation of transport proteins (ferritin and transferrin). So, the toxic NTBI can generate reactive radicals via Fenton reactions, resulting in oxidative organ damage^40^. These results are in accordance with the previous study that reported iron overload elicited a significant increase in all iron indices^41^. The increased ferritin level in iron-overloaded mice may contribute to an excessive iron deposition since ferritin is an intracellular protein responsible for iron storage in a non-toxic form and reduced cellular oxidative damage mediated by iron^42^.

Further, iron overload syndrome is characterized by ineffective hematogenesis, leading to excess iron deposition throughout the body^43^. According to the present findings, excess iron caused the altered erythropoiesis as indicated by a significant (P < 0.05) increment in total RBCs, Hb, and hematocrit of IOL mice, suggesting that increased iron absorption influences bone marrow function. This finding is consistent with the previous studies in which mice administrated with excess iron showed abnormal RBC indices^44,45^. Also, the current study revealed that excess iron induced the expanded RBC size and higher cellular hemoglobin concentrations, as confirmed by a significant (P < 0.05) increment in MCV and MCH of IOL mice. This increase may be a way of sequestering iron into a non-toxic compartment, as Li et al.^46^ suggested. Furthermore, the current study recorded a significant (P < 0.05) increase in total WBCs and decreased platelet count in IOL mice, suggesting that iron overload influences the hematopoietic system. Chai et al.^45^ demonstrated that hematopoiesis may be established in extra-medullary sites, prominently in the spleen, when bone marrow function is altered, resulting in ineffective hematopoiesis. Thus, the results confirmed that iron overload may lead to extra-medullary hematopoiesis, as Chai et al.^45^ stated. Additionally, the high augmented level of the total WBCs in IOL mice may contribute to enhanced immune systems and organ-specific inflammation since iron overload can aggravate the chances of infection, and this postulation is supported by Habib et al.^47^.

Indeed, the current study exhibited that iron accumulation caused increased relative liver weight, which is in line with previous studies^37,48^. Gao et al.^37^ reported that distinct hepatic iron deposition induced hepatocyte damage and disturbed hepatic function, which likely stimulated hepatocellular inflammation as well as edema and consequently increased the relative liver weight. The ongoing study revealed that hepatic iron accumulation results in the leakage of cellular enzymes into the bloodstream, leading to increased levels of AST and ALT, confirming hepatocellular damage. The leakage of cellular enzymes may be clearly due to the increased liver membrane permeability, as Talaei et al.^49^ mentioned. Additionally, Azab et al.^41^ attributed the increased transaminase activities to the generation of ROS and oxidative damage by excess hepatic iron and, subsequently, hepatic damage. Moreover, chronic iron load elicits significant damage in hepatic parenchyma, resulting in the failure of normal uptake, conjugation, excretion, and subsequent decrease in total protein and albumin content, as Al Basher^50^ stated. These results are in harmony with the histological study, which demonstrated substantial changes in hepatic tissue, including hepatocellular necrosis and apoptosis, mononuclear cell infiltration, and diffused Kupffer cell hyperplasia. This is consistent with previous reports showing iron accumulation induces hepatic damage^37,50^.

Moreover, the increased iron accumulation can cause renal damage, as indicated by a marked increase in the relative kidney weight and a significant (P < 0.05) elevation in the creatinine level in IOL mice. This finding is accompanied by histological findings that revealed extensive alterations in renal tissue, including glomerular degeneration, glomerular basement membrane thickening, mesangial matrix expansion, and tubular degeneration. These renal structure changes probably contributed to the increased relative kidney weight, as Mahajan et al.^51^ suggested. The renal function disturbance is similar to the previous study, which revealed iron overload caused the elevated creatinine level^52^. The increment in serum creatinine may be attributed to the destruction and malfunction of nephron tubule cells that contain the receptors and transporters related to iron hemostasis^53^. The destruction of tubule cells may be due to excess iron induced over-expression of nitric oxide, which combines with superoxide anions to form peroxynitrite, mediating lipid peroxidation and oxidative damage to tubule cellular membrane^54^.

Free iron interacts with H_2_O_2_ to form hydroxyl radical (HO^**·**^) through the Fenton reaction, which further initiates the chain reaction and generates other ROS^55^. Also, excess liver iron may cause peroxidative injury to membrane phospholipids, forming lipid peroxides that promote hepatic and renal damage^55^. Thus, oxidative damage is considered the underlying mechanism for the detrimental effects of iron on hepatic and renal tissue. As demonstrated in the current study, iron administration increased oxidative stress status in both the liver and kidney of mice and this may be due to one or more causes of the following: (i) a significant increase in serum iron; (ii) a significant rise in lipid peroxidation products (MDA); (iii) a marked decrease in GSH due to enhancement in its consumption; (iv) a dramatic decrease in the activity of antioxidant enzymes (SOD, CAT, and GR) in hepatic and renal tissues indicating hepatorenal oxidative damage. Such alterations may result from the increased NTBI that can be catalyzed and produce ROS, especially highly toxic free hydroxyl radicals, via Fenton reactions that may finally result in chronic hepatorenal diseases^56^. In addition, hepatic and renal mitochondria were also damaged by increased oxidative stress and mitochondrial depolarization in IOL mice. Since mitochondria are potential organelles responsible for iron hemostasis, iron-mediated oxidative stress causes hepatic and renal mitochondrial damage and dysfunction, leading to impaired hepatorenal dysfunction. The mitochondria membrane alterations evoke ROS generation via increased lipid peroxidation and, subsequently, could change the fluidity of the inner mitochondrial membrane and subsequent mitochondrial depolarization, as Liu et al.^57^ demonstrated. Moreover, Liu et al.^8^ clarified that the increase in ROS reaches a threshold level activating the mitochondrial permeability transition pore (mPTP) opening, which in turn leads to the simultaneous collapse of Δψ and a permanent increase in ROS production by the electron transfer chain, resulting in a decrease in cell viability. Collectively, these results support that iron accumulation impaired hepatic and renal function via increased oxidative stress and mitochondrial dysfunction. These findings are compatible with the previous studies, which revealed iron overload-mediated free radicals stress can result in cellular and mitochondrial dysfunction^58–60^.

Besides oxidative stress, apoptotic cell death is regarded as one of the operating mechanisms in iron overload-mediated hepatorenal damage via cell DNA fragmentation^50^. The current study reported that excess iron mediating oxidative stress synergizes in promoting strand cleavage of nuclear DNA, conferring fragmentation in hepatic and renal cells, indicating hepatic and renal cell necrosis or apoptosis. Similarly, several previous studies demonstrated DNA damage following excess iron deposition in various tissues^50,61,62^. The recorded DNA fragmentation in hepatorenal tissue may be attributed to the overproduction of ROS caused by excess iron accumulation, mitochondrial dysfunction, and decreased antioxidant status^61^. Additionally, nuclear DNA damage may be due to the suppression of SOD activity resulting in the accumulation of superoxide radicals which promotes the hydroxyl-radical formation and subsequently attacks the adjacent DNA^63^. Furthermore, mitochondrial DNA is more susceptible to oxidative alterations in its coding region, where mitochondrial DNA is found near the inner mitochondria membrane, which is the vital site for ROS generation, leading to damage to mitochondrial DNA^64^. The present study revealed that excess iron caused mitochondrial DNA damage, as confirmed by a marked decrease in mitochondrial DNA copies in IOL mice. The recorded excess iron induced a dramatic decrease in the number of mitochondrial DNA copies, which could also be explained by the aforementioned iron overload-induced increased intracellular ROS generation, as Mohamed et al.^64^ stated.

Dietary intake of Chia or CoQ_10_ administration effectively reduces iron accumulation in the blood by 54.47% and 54.53%, respectively. In comparison, combined administration of both decreases serum iron levels by 56.55%, suggesting their ability to chelate iron effectively. This result was ensured by the in vitro study, which proved that Chia and CoQ_10_ could bind free iron. In addition, the in silico study displays the lowest conformation minimum energy (− 1,462,194 kcal/mol) and the lowest dielectric energy (− 3167 kcal/mol) of phytic acid. This indicates the high stability of phytic acid compared to deferoxamine and CoQ_10_, which reflects its usefulness as an iron chelator inside the cell. Also, the largest number of Hydrogen-bond donors (12) and acceptors (24), the lowest ionization potential (− 11.567 eV), and the lowest molar refractivity value (97.571) of phytic acid indicate the high reactivity of phytic acid compared to the other two compounds. Additionally, phytic acid shows a log P value of 2.303, demonstrating its ability to pass through the cell membrane easily. Further, phytic acid shows the best thermodynamic properties compared to deferoxamine and CoQ_10_. It has the lowest average free energy, average enthalpy, average entropy, average heat capacity, and average heat of formation. These values reflect the higher stability of phytic acid compared to the other two compounds.

Furthermore, decreased levels of ferritin, transferrin, and TIBC in IOL mice treated with Chia or CoQ10 indicate the maintenance of iron hemostasis. Hence, Chia or CoQ_10_ can effectively prevent iron deposition in the blood via the suppression of ferritin and transferrin expression, as Bhowmik et al.^65^ postulated. The current study attributed the chelating property of Chia to its phytic acid content and hydroxyl groups of its polyphenols that can chelate iron and enhance its excretion, resulting in decreased iron accumulation in tissues. This hypothesis aligns with previous reports by Oliveira-Alves et al.^66^ and Rahman et al.^67^. The ability of CoQ_10_ to decrease iron indices may be the rationale for its antioxidant property rather than its high lipophilicity, which allows it to move freely across cellular barriers, facilitating the removal of toxic metals from various organs^68,69^.

Treatment with Chia and CoQ_10_ caused a significant (P < 0.05) amelioration in the hematogenesis process, as indicated by restoring the RBCs near the corresponding control value. These results were in line with the previous studies in which dietary supplementation with Chia in rats fed a high-fat diet, and CoQ_10_ administration improved the RBCs, Hb, MCV, and MCH^70,71^. The ongoing study postulated that Chia improved erythropoiesis in IOL mice due to its nutritional content of protein, minerals, and vitamins that reinforce erythropoiesis^72^. Additionally, the modulated erythropoiesis in IOL mice administrated CoQ_10_ may be attributed to its antioxidant activity that reduced the susceptibility of RBCs to membrane oxidative damage, subsequently resulting in the maintenance of the normal Hb levels as Gitgona et al.^73^ declared. Remarkably, the current results demonstrated the stabilized total WBCs in IOL mice supplemented with Chia or CoQ_10,_ suggesting their ability to suppress immune-stimulatory lymphoproliferative responses resulted from iron overload. This ability of Chia and CoQ_10_ may be due to their antioxidant and anti-inflammatory components acting locally as cytokines and defending the body against infection. This subsequently leads to a decrease in the total WBC level, as Nesaretnam et al.^74^ reported. The current findings are supported by previous studies^73,75^.

Regarding hepatorenal damage, administration of Chia and CoQ_10_ could alleviate hepatorenal damage induced by iron overload, suggesting protection by maintaining the structural integrity of the hepatocellular and nephron tubule cellular membranes. This is implied by a marked decrease in relative liver and kidney weights, hepatocellular enzyme activities, and creatinine level. Also, these treatments restored hepatic synthetic function as indicated by a significant (P < 0.05) increment in total protein and albumin content. These findings correlated with histological results, revealing restored hepatic and renal structure. Similar findings were revealed by the previous reports, which reported hepatorenal protective potency of CoQ_10_ and SHS in different hepatic and renal damage models^76–78^. This study expected that the hepatorenal protective potency of Chia and CoQ_10_ may be due to their ability to chelate and scavenge iron, reduce oxidative damage, and restore redox balance by preventing ROS generation. Since the antioxidant ability could form a stabilized phenoxy radical that can effectively scavenge excess iron and counteract hepatorenal damage^79^. Moreover, the current study supposed that CoQ_10_ may initiate the antioxidant defense mechanism and prevent the inflammatory and apoptosis events in response to renal damage caused by iron overload, as Al-Megrin et al.^80^ demonstrated.

Thereby, the present study suggested that the mechanism underlying the alleviating potency of Chia and CoQ_10_ against iron overload may involve the antioxidant and iron chelating properties of Chia and CoQ_10_. This assumption is supported by their ability to diminish MDA content, increase GSH level, and significantly boost antioxidant CAT, SOD, and GR enzymatic activities in both hepatic and renal tissues. The antioxidant potency of Chia is mainly attributed to its bioactive constituents, including phenolic compounds, which have hydroxyl groups that are readily oxidized to produce the corresponding effective scavengers of ROS called *O*-quinones^81^.; the antioxidant potency of CoQ_10_ may be attributed to its ability to potentiate electron transport chain where CoQ_10_ plays a vital role in electron donor and acceptor in the hepatic and renal mitochondria as Song et al.^82^ suggested. Further, the decreased MDA content in IOL mice treated with CoQ_10_ may be due to its ability to maintain the membrane fluidity, which protects membranous phospholipids against peroxidation ^83^. In addition, the increased SOD and CAT in Chia and CoQ_10_ explained the hepatorenal protection, as SOD converts the harmful superoxide radicals into oxygen and hydrogen peroxide, followed by CAT, which detoxifies the toxic hydrogen peroxide into water and oxygen^84^. Additionally, Chia and CoQ_10_ induced marked amelioration in hepatic and renal mitochondrial damage induced by IOL, resulting in a subsequent decrease in apoptotic cell death. Thus, the current study suggested that Chia and CoQ_10_ also have a pronounced anti-apoptotic activity against the apoptosis progression of hepatic and renal cells in IOL mice, as demonstrated by a significant decrease in hepatic and renal DNA fragmentation. This suggestion is consistent with previous studies^75,85^. The antioxidant mechanism of Chia results in suppressing oxidative DNA and mitochondrial damage^86^. Moreover, Souri et al.^87^ suggested that the reduced DNA damage after CoQ_10_ supplementation is attributed to enhanced DNA repair enzyme activity. Another possible mechanism of hepatorenal ameliorative potency of Chia and CoQ_10_ is increasing the mitochondrial DNA copies in hepatorenal tissue. Upon supplementation with Chia and CoQ_10_, a significant increase in mitochondrial DNA copies was recorded in the hepatic and renal tissue of IOL mice, indicating the maintenance of oxidative/antioxidant balance.

## Conclusion

The present study revealed that iron exerts its toxicity on hematopoietic and hepatorenal tissue via initiating and propagating several ROS leading to oxidative damage, mitochondrial damage, and DNA fragmentation, which mediates apoptosis via changes in the number of mitochondrial DNA copies in hepatic and renal cells. Remarkably, Chia can chelate free iron via its phytic acid component in the hepatic and renal cells, and CoQ_10_ can scavenge free radicals. Combining Chia (iron chelator) and CoQ_10_ (antioxidant) may show a synergistic effect over monotherapy against iron overload. This is revealed by diminishing ROS, mitochondrial damage, and DNA cleavage, and maintains iron homeostasis.

## Data Availability

The datasets used and/or analyzed during the current study are available from the corresponding author on reasonable request.

## Abbreviations

IOL: Iron overload
CoQ_10_: Coenzyme Q_10_
ROS: Reactive oxygen species
Log P: Partition coefficient
HOMO: Highest occupied molecular orbital
LUMO: Lowest unoccupied molecular orbital
EC_50_: Half-maximal effective concentration
IPC: Ferric hydroxide poly maltose complex
TIBC: Total iron-binding capacity
TSI: Transferrin saturation index
UIBC: Unsaturated iron-binding capacity
RBCs: Red blood cells
Hb: Hemoglobin
HCT: Hematocrit
MCV: Mean corpuscular volume
MCH: Mean corpuscular hemoglobin
MDA: Malondialdehyde
GSH: Glutathione reduced
SOD: Superoxide dismutase
CAT: Catalase
GR: Glutathione reductase
MMP: Mitochondrial membrane potential change
PBS: Phosphate buffer saline
TCA: Trichloroacetic acid
mPTP: Mitochondrial permeability transition pore
